# Low expression of TRAF3IP2-AS1 promotes progression of *NONO-TFE3* translocation renal cell carcinoma by stimulating *N*^6^-methyladenosine of PARP1 mRNA and downregulating PTEN

**DOI:** 10.1186/s13045-021-01059-5

**Published:** 2021-03-19

**Authors:** Lei Yang, Yi Chen, Ning Liu, QianCheng Shi, Xiaodong Han, Weidong Gan, Dongmei Li

**Affiliations:** 1 Immunology and Reproduction Biology Laboratory & State Key Laboratory of Analytical Chemistry for Life Science, Medical School, Nanjing University, Nanjing, 210093 Jiangsu China; 2 Jiangsu Key Laboratory of Molecular Medicine, Nanjing University, Nanjing, 210093 Jiangsu China; 3 Department of Urology, Affiliated Drum Tower Hospital of Medical School of Nanjing University, Nanjing, 210008 Jiangsu China

**Keywords:** TRAF3IP2-AS1, M^6^A modification, NONO-TFE3, PARP1, PTEN

## Abstract

**Background:**

*NONO-TFE3* translocation renal cell carcinoma (*NONO-TFE3* tRCC) is one subtype of RCCs associated with Xp11.2 translocation/TFE3 gene fusions RCC (Xp11.2 tRCCs). Long non-coding RNA (lncRNA) has attracted great attention in cancer research. The function and mechanisms of TRAF3IP2 antisense RNA 1 (TRAF3IP2-AS1), a natural antisense lncRNA, in *NONO-TFE3* tRCC remain poorly understood.

**Methods:**

FISH and qRT-PCR were undertaken to study the expression, localization and clinical significance of TRAF3IP2-AS1 in Xp11.2 tRCC tissues and cells. The functions of TRAF3IP2-AS1 in tRCC were investigated by proliferation analysis, EdU staining, colony and sphere formation assay, Transwell assay and apoptosis analysis. The regulatory mechanisms among TRAF3IP2-AS1, PARP1, PTEN and miR-200a-3p/153-3p/141-3p were investigated by luciferase assay, RNA immunoprecipitation, Western blot and immunohistochemistry.

**Results:**

The expression of TRAF3IP2-AS1 was suppressed by NONO-TFE3 fusion in *NONO-TFE3* tRCC tissues and cells. Overexpression of TRAF3IP2-AS1 inhibited the proliferation, migration and invasion of UOK109 cells which were derived from cancer tissue of patient with *NONO-TFE3* tRCC. Mechanistic studies revealed that TRAF3IP2-AS1 accelerated the decay of PARP1 mRNA by direct binding and recruitment of *N*^6^-methyladenosie methyltransferase complex. Meanwhile, TRAF3IP2-AS1 competitively bound to miR-200a-3p/153-3p/141-3p and prevented those from decreasing the level of PTEN.

**Conclusions:**

TRAF3IP2-AS1 functions as a tumor suppressor in *NONO-TFE3* tRCC progression and may serve as a novel target for *NONO-TFE3* tRCC therapy. TRAF3IP2-AS1 expression has the potential to serve as a novel diagnostic and prognostic biomarker for *NONO-TFE3* tRCC detection.

**Supplementary Information:**

The online version contains supplementary material available at 10.1186/s13045-021-01059-5.

## Background

Xp11.2 translocation renal cell carcinoma (Xp11.2 tRCC), also known as transcription factor binding to IGHM enhancer 3 (*TFE3*)-fusion associated RCC, is recently stratified in the MiT (microphthalmia transcription factor) family tRCC as a new subset of RCC in the 2016 WHO classification [1]. Xp11.2 tRCC, which features *TFE3* fusion gene and poor prognosis of patients, is caused by several different translocations involving chromosome *TFE3* with partner genes, including *ASPL*, *PRCC*, *NONO* and *CLTC*, as well as some other housekeeping genes [2–6]. The fusion of *NONO* to *TFE3*, which is caused by X chromosome inversion inv(X)(p11.2;q12)[5], contains 1–7 exons of *NONO* and 6–10 exons of *TFE3* [7]. TFE3-fusion proteins commonly retain the DNA binding domain (9–10 exons of wild type *TFE3*), indicating that these overexpressed TFE3-fusion proteins can function as oncogenic transcription factors. Accumulating evidence suggests that signaling pathways or proteins well implicated in carcinogenesis are regulated by the TFE3 protein in part, including E-cadherin, mTORC1 signaling and folliculin signaling [8–10].

Long non-coding RNAs (lncRNAs) are a newly discovered heterogeneous class of transcripts larger than 200 nucleotides and limited protein-coding potential [10]. Recent research has reported that lncRNAs participate in diverse physiological and pathological processes, such as cell cycle, cell differentiation and metastasis [11]. A wide range of expression levels and distinct cellular distributions are characteristic features of lncRNAs, and thus, lncRNAs could function as a large and diverse class of regulators. For example, lncRNAs function as competing endogenous RNA (ceRNA) to regulate post-transcriptionally expression target gene indirectly through absorbing miRNAs in the cytoplasm [12], or to directly bind to or physically interact with target mRNA, leading to regulation of translation or mRNA decay [13–15]. However, the potential molecular mechanism of lncRNAs associated with the oncogenesis of Xp11.2 tRCC is still unclear.

In our previous study, the chromatin immunoprecipitation sequencing (ChIP-seq) data reveal that NONO-TFE3 can bind to promoter region of TRAF3IP2 antisense RNA 1 (TRAF3IP2-AS1) [16]. TRAF3IP2-AS1, as a natural antisense lncRNA, is expressed from *TRAF3IP2*. As a member of the TNF receptor associated protein, TRAF3IP2 is believed to be involved in inflammation and tumor development. Through binding to IL-17 mRNA directly and promoting the stability of mRNA, TRAF3IP2 enhances inflammation in tumor cells [17]. Other study also reveals that TRAF3IP2 enhances tumor growth of glioblastoma by promoting inflammation of microenvironment and suppressing tumor angiogenesis [18]. Although the results of bioinformatics reported currently indicate that the abnormal expression of TRAF3IP2-AS1 in glioblastoma could be a biological marker of tumor progression [19], the specific biology function of TRAF3IP2-AS1 and the actual mechanism is still unknown. Therefore, the role and mechanisms of TRAF3IP2-AS1 in Xp11.2 tRCC need to be addressed, which may provide therapeutic and prognostic value for Xp11.2 tRCC.

Herein, we found that NONO-TFE3-fusion protein negatively regulated TRAF3IP2-AS1 and upregulation of TRAF3IP2-AS1 could inhibit cell proliferation, migration and invasion in *NONO-TFE3* tRCC*.* Mechanistically, we found that TRAF3IP2-AS1 directly interacted with poly ADP-ribose polymerase (PARP1) mRNA, a known oncogene, to promote the mRNA decay by stimulating m^6^A modification, but, in *NONO-TFE3* tRCC, low expression of TRAF3IP2-AS1 caused upregulation of PARP1 by decreasing the PARP1 mRNA decay. In addition, TRAF3IP2-AS1 could function as a ceRNA to modulate the expression of phosphatase and tensin homolog (PTEN) through post-transcriptional regulation of miRNAs. Therefore, our results indicate that TRAF3IP2-AS1 plays a critical role in tumor progression as a tumor suppressor in *NONO-TFE3* tRCC, which highlights a novel regulatory mechanism underlying tumorigenesis and tumor development.

## Methods

### Cell culture

HEK293T, HK-2 and 786-O cell lines were purchased from Type Culture Collection of Chinese Academy of Sciences (Shanghai, China), and the Xp11.2 tRCC cell lines UOK120 and UOK109 were kindly provided by Dr. W. Marston Linehan (National Cancer Institute, Bethesda, MD). The UOK120 (*PRCC-TFE3* fusion) and UOK109 (*NONO-TFE3* fusion) cell lines were derived from primary papillary cell carcinoma as described [20], and were derived from tumors arising in a 30- and a 39-year-old male, respectively. Cells were maintained in DMEM (Gibco, Grand Island, NY) supplemented with 10% FBS (Gibco) and 1% penicillin–streptomycin (Invitrogen, Carlsbad, CA). Cells were cultured in a standard humidified atmosphere of 5% CO_2_ at 37 °C.

### Tissue samples

All the tissue samples were obtained from Nanjing Drum Tower Hospital and confirmed by a senior pathologist (Department of Pathology, Nanjing Drum Tower Hospital). All patients were informed that their tissues will be used into scientific research.

### RNA isolation and quantitative real-time PCR (qRT-PCR) assays

Total RNA was isolated using Trizol reagent (Invitrogen) according to the product description. Cytoplasmic and nuclear RNA was extracted using an RNA Purification Kit (Norgen Biotek, Thorold, ON, Canada) according to the manufacturer’s instructions. RNA was reverse-transcribed into cDNA using Hiscript Q RT Super-mix for qPCR (Vazyme, Nanjing, China), cDNA was quantified by qRT-PCR, and the data were acquired with ChamQ SYBR qPCR Master Mix (Vazyme). 18 s rRNA was chosen as internal control for normalization. The level of 18 s rRNA was also quantified to confirm the relative expression of TRAF3IP2-AS1. The expression level of miRNAs was detected by miRNA Universal SYBR qPCR Master Mix (Vazyme). Single-stranded cDNA was synthesized by using miRNA 1st Strand cDNA Synthesis Kit (Vazyme). The U6 snRNA was used for loading control. The primers for RNAs are shown in Additional file 1: Table S1.

### ChIP assay and dCas9-ChIP assay

ChIP assay and dCas9-ChIP assay [21] were performed according to the protocol of a Pierce™ Agarose ChIP Kit (Thermo Scientific, Carlsbad, CA) to assess binding ability of NONO-TFE3 to TRAF3IP2-AS1 promoter. Briefly, after indicated treatment, the cells were fixed, lysed and sonicated to appropriate fragments. The prepared chromatin was precipitated using specific antibodies overnight. Then, the binding complexes were thoroughly washed, eluted, purified and analyzed by qRT-PCR or western blot. Primer sets targeting those regions containing potential NONO-TFE3 binding sites in TRAF3IP2-AS1 promoter are provided in Additional file 1: Table S2.

### Dual-luciferase reporter assay

HEK293T cells were seeded in 24-well plates and transfected with pmirGLO-PARP1-3′UTR plasmid. The firefly luciferase and Renilla luciferase activity in each cell were detected by Dual Luciferase Reporter Assay Kit (Vazyme). HEK293T cells were transfected with pGL3-Basic-TRAF3IP2-AS1 vector, after which promoter luciferase activity was measured. Moreover, pRL-TK-Renilla luciferase reporter construct was used as an internal control.

### RNA immunoprecipitation (RIP) analyses

RIP assays were performed according to the instructions of a Millipore Magna RIP Kit (Millipore, Darmstadt, Germany). The indicated cells were lysed with RIP lysis buffer containing protease and RNase inhibitor, after which cell lysate supernatant was incubated with anti-GFP/AGO2/YTHDF1/YTHDF2/IgG antibody-conjugated beads overnight at 4 °C. Then, the binding complexes were thoroughly washed, eluted, purified and analyzed by qRT-PCR.

### m^6^A RIP analyses

Magna MeRIP™ m^6^A kit was chosen to assess m^6^A modification levels in target mRNA according to the manufacturer’s instructions (Millipore). Briefly, the isolated RNA needs to be fragmented by RNA fragmentation buffer. After saving one-tenth of the total RNA as input, the remaining RNAs were performed for immunoprecipitation with m^6^A antibody coated on magnetic beads A/G. Then, the binding complexes were thoroughly washed, eluted, purified and analyzed by qRT-PCR. Specific primer information about PARP1 is listed in Additional file 1: Table S3.

### Western blot

Total protein was isolated from cells following various treatments. Cells were washed three times with PBS and lysed in ice cold extraction buffer. After centrifuged, soluble fractions were mixed with 5 × loading buffer and heated at 100 °C for 5 min. Proteins were separated using SDS-PAGE and the PVDF membrane (Roche, Basel, Switzerland) by standard procedures. Blots were blocked for 1 h at room temperature in TBS with 0.05% Tween 20 (Sigma-Aldrich, St Louis, MO) and 5% nonfat milk. Primary antibodies were incubated overnight at 4 °C in Tris Buffered Saline Tween (TBST) with 3% BSA (Sigma-Aldrich). HRP-conjugated secondary antibodies were incubated 1 h at room temperature. Protein signals were detected using ECL solution (Millipore), and band intensities were quantified using Image J software (National Institutes of Health). Additionally, ACTB was chosen as internal control.

### Flow cytometry

Flow cytometry was performed according to the manufacture’s protocol. After incubation with reagents from an Annexin V-FITC/Propidium Iodide (PI) Apoptosis Kit (BD Biosciences, Franklin Lakes, NJ), cells were analyzed using a BD Beckman cytometer (BD Biosciences) and FlowJo software. For cell cycle analysis, cells were incubated with reagents from a PI/RNase Staining Kit (BD Biosciences). Then, the cells were analyzed on a BD Beckman cytometer.

### CCK8, 5-Ethyny-2′-deoxyuridine (EdU) assay and clone forming

The indicated cells were seeded in the plates. Cell proliferation assay was performed using the Cell Counting Kit 8 (CCK8; Vazyme). EdU (Beyotime, Shanghai, China) was operated according to the manufacture’s protocol. Transfected cells were seed into 6-well plate with 500 cells/well for 10–14 days to assess the clone-forming capacity.

### Transwell assay

Cell migration and invasion assays were performed using Transwell technique with uncoated polycarbonate inserts (Millipore) for migration or BioCoat™ inserts (BD Biosciences) for invasion. Medium without FBS (1–5 × 10^4^ cells/200 μL) was added into the upper chamber, with 500 μL DMEM containing 10% FBS added into the lower chamber. After crystal violet staining, the positive cells were counted and analyzed under microscope.

### Immunohistochemistry (IHC)

Paraffin-embedded sections were firstly deparaffinized and then incubated with rabbit polyclonal anti-TFE3 (Sigma-Aldrich) primary antibody at 4 °C overnight. After three times wash by PBST, the sections were then incubation with HRP conjugated goat anti-mouse or goat anti-rabbit secondary antibody. The sections were washed by PBST for three times, and the signal was detected using DAB Substrate Kit following the manufacture’s instruction.

### Fluorescence in situ hybridization (FISH)

Cy3-labeled TRAF3IP2-AS1 probes were synthesized by GenePharma Technology (Shanghai, China). FISH was performed using a FISH Kit (GenePharma) according to the manufacturer’s instructions. Nuclei were stained with DAPI. Images were acquired on a FV3000 confocal fluorescence microscope. The sequences are provided in Additional file 1: Table S4.

### Sphere formation

For sphere formation assay, proper cells were seeded in ultra-low attachment 96-well plates and cultured in DMEM supplemented with 10% FBS. The sphere pictures were taken 2 weeks later. For sphere formation assay, 1000 UOK109 or 786-O cells were used. Ultra-low attachment plates (cat. no. 174925) were purchased from Corning Incorporated (Corning, NY).

### Plasmid construction, small interfering RNA (siRNA), antisense oligonucleotides (ASOs), lentivirus and cell transfection

The human TRAF3IP2-AS1 sequence was synthesized by GeneChem Technology (Shanghai, China) and subcloned into pcDNA3.1 and pSL-MS2-12x (Addgene, Cambridge, MA). CRISPR/Cas9-based Synergistic Activation Mediator (SAM) system [22] was constructed by GeneChem Technology. Targeted RNA methylation system was constructed by standard procedures including enzyme digestions, PCR and subcloning according to Hao Du’s [23], Jiexin Li’s [24] and Christopher Wilson’s [25] protocol. SiRNA was synthesized by Gene Pharma (Suzhou, China), and ASOs were synthesized by RiboBio (Guangzhou, China). A mixed-modality approach combining ASOs and RNAi reagents improved knockdown efficacy [26], because TRAF3IP2-AS1 localizes in both nuclear and cytoplasmic compartments. Lentivirus, TFE3-shRNA, was synthesized by OBiO Technology (Shanghai, China). Cells were transfected with siRNAs or plasmids using LipoFiter 3.0 (Hanbio, Shanghai, China) according to the manufacturer’s instructions. Treatments were administered 24 h after transfection. Cells were harvested 48 h after transfection. The sequences are provided in Additional file 1: Table S5-6.

### Statistical analysis

Statistical analyses were performed using SPSS 22.0 software (SPSS Inc., Chicago, IL). GraphPad Prism 8.0 (GraphPad Software, San Diego, CA) was applied to plot the data. Student’s t test and one-way analysis of variance (*ANOVA*) were used to assess the significance of differences. *P* < 0.05 was considered statistically significance (*P < 0.05, ** *P* < 0.01, and *** *P* < 0.001). All values are expressed as the means ± standard deviation.

## Results

### TRAF3IP2-AS1 expression is significantly decreased in *NONO-TFE3* tRCC

**Fig. 1:**
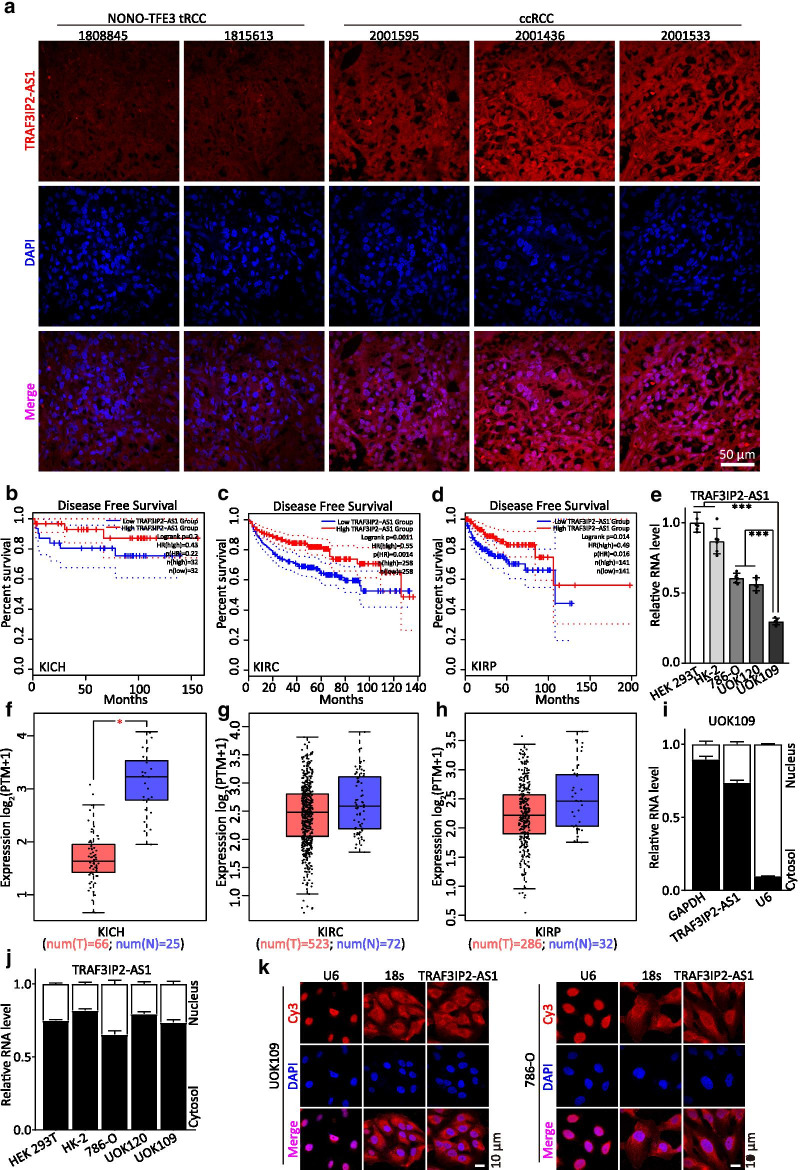
TRAF3IP2-AS1 expression is significantly decreased in *NONO-TFE3* tRCC. **a** The RNA level of TRAF3IP2-AS1 (red) was analyzed by FISH assays in *NONO-TFE3* tRCC and ccRCC. DAPI-stained nuclei are blue. **b–d** Kaplan–Meier analysis revealed the disease-free survival (DFS) in KICH, KIRC and KIRP patients based on the relative TRAF3IP2-AS1 expression. **e** The RNA level of TRAF3IP2-AS1 was analyzed by qRT-PCR assay in ccRCC cell line (786-O), tRCC cell lines (UOK109 and UOK120) and normal cell lines (HK-2 and HEK293T). **f–h** Analysis of TRAF3IP2-AS1 in KICH, KIRC and KIRP tissues compared with normal tissues were performed using TCGA data. **i–j** The subcellular distribution of TRAF3IP2-AS1 was analyzed via qRT-PCR in HEK293T, HK-2, 786-O, UOK120 and UOK109 cells. U6 and GAPDH were used as nuclear and cytoplasmic markers, respectively. **k** The location of TRAF3IP2-AS1 (red) in UOK109 and 786-O cells was determined by FISH assay. U6 and 18s rRNA were used as positive controls for the nuclear and cytoplasmic fractions, respectively. DAPI-stained nuclei are blue. The data are presented as the mean ± SD, *P < 0.05, ***P < 0.001

In our previous study, *TRAF2IP2-AS1* was identified as a potential target gene of NONO-TFE3 by ChIP-seq [16]. To explore if TRAF3IP2-AS1 expression was linked to *NONO-TFE3* tRCC, the expression and distribution of TRAF3IP2-AS1 were detected by FISH. The results showed low level expression of TRAF3IP2-AS1 in *NONO-TFE3* tRCC compared with that in other clear cell renal cell carcinoma (ccRCC) samples (Fig. 1a). The relationships between TRAF3IP2-AS1 expression and the prognosis of patients with indicated cancer were investigated using the GEPIA database (http://gepia.cancer-pku.cn/) [27]. These results demonstrated a significant correlation between low expression of TRAF3IP2-AS1 and poor prognosis in kidney chromophobe (KICH), kidney renal clear cell carcinoma (KIRC) and kidney renal papillary cell carcinoma (KIRP; Fig. 1b–d). The expression of the TRAF3IP2-AS1 in tumor groups was lower than that of normal groups, especially in KICH (Fig. 1f–h).

To investigate the role of TRAF3IP2-AS1 in *NONO-TFE3* tRCC progression, we first evaluated TRAF3IP2-AS1 expression levels in various RCC cell lines and normal cell. The highest expression of TRAF3IP2-AS1 was observed in HEK293T cell line, whereas the lowest level was detected in UOK109 cell line which was derived from tumor tissue of patient with *NONO-TFE3* tRCC (Fig. 1e). TRAF3IP2-AS1 expression showed both nuclear and cytoplasmic localization in the UOK109 cells; however, cytoplasmic expression was generally predominant (Fig. 1i). The same phenomena were observed in HEK293T, HK-2, 786-O and UOK120 cell lines (Fig. 1j). In addition, the result of FISH showed that TRAF3IP2-AS1 was located in the cytoplasm in UOK109 and 786-O cells (Fig. 1k). Taken together, the above data revealed that TRAF3IP2-AS1 was low-expressed in the cytoplasm of *NONO-TFE3* tRCC.

### Low expression of TRAF3IP2-AS1 accelerates development of *NONO-TFE3* tRCC

**Fig. 2:**
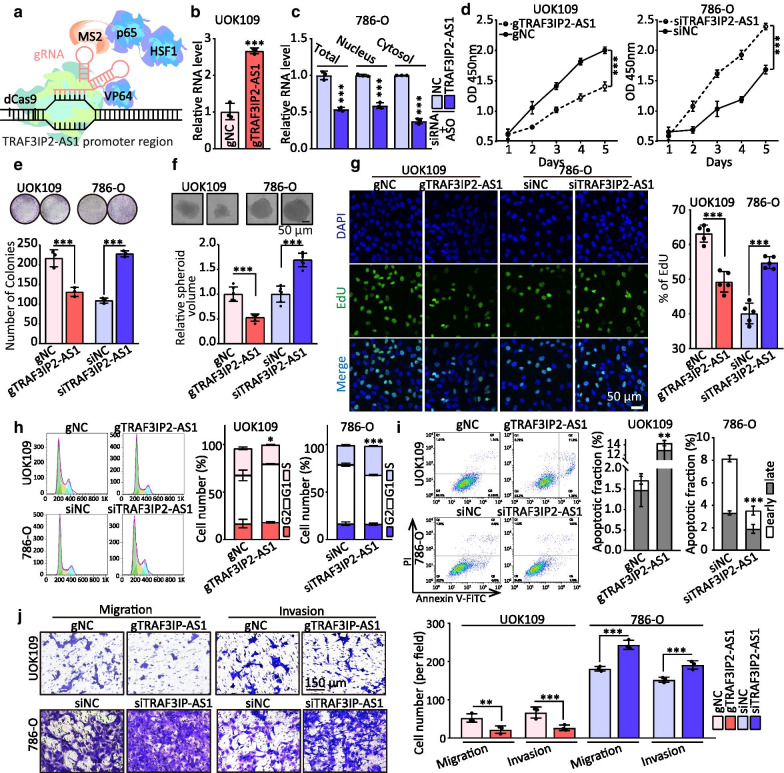
TRAF3IP2-AS1 deficiency induces development of *NONO-TFE3* tRCC. **a** Schematic illustration of CRISPR/Cas9-based Synergistic Activation Mediator (SAM) system. **b** The RNA level of TRAF3IP2-AS1 was analyzed by qRT-PCR assay in UOK109 transfected with dCas9 and guide RNA targeting TRAF3IP2-AS1 promoter (gTRAF3IP2-AS1). **c** The RNA level of TRAF3IP2-AS1 was analyzed by qRT-PCR assay in 786-O transfected with siRNA and antisense oligonucleotides (ASOs). **d–f** The effects of TRAF3IP2-AS1 overexpression or knockdown on the proliferation of UOK109 and 786-O cells, respectively, were examined by CCK-8 assay (**d**), colony formation assays (**e**) and tumor sphere formation (**f**). **g** EdU assays were used to detect the proliferation rate of UOK109 and 786-O cells after transfection for 48 h. **h** Cell cycle was analyzed using flow cytometry after transfection for 48 h. **i** Cell apoptosis was analyzed via flow cytometry using an Annexin V/PI Kit after transfection for 48 h. **j** Migration and invasion assays were performed with transfected cells using Transwell inserts. The data are presented as the mean ± SD, *P < 0.05, **P < 0.01, ***P < 0.001

To determine the functional role of TRAF3IP2-AS1 in biological behavior of *NONO-TFE3* tRCC, the effects of downregulated or upregulated TRAF3IP2-AS1 on cancer cell growth were investigated. SAM system was applied to up-regulate endogenous TRAF3IP2-AS1, and three siRNAs and ASOs were designed to decrease expression of TRAF3IP2-AS1 in the cytoplasm or in the nucleus, respectively (Fig. 2a–c). The results showed that cell proliferation, colony formation and tumor sphere formation were inhibited by TRAF3IP2-AS1 overexpression compared with the negative control in UOK109 cells, and knockdown of TRAF3IP2-AS1 significantly increased cell proliferation, colony formation and tumor sphere formation in 786-O cell line (Fig. 2d–f).

Additionally, an EdU assay showed a significant inhibition of cell proliferating activity caused by overexpression of TRAF3IP2-AS1 in UOK109 cells, and opposite results were observed in 786-O cells with lowering expression of TRAF3IP2-AS1 (Fig. 2g), which was confirmed by cell cycle assay (Fig. 2h), and was validated by testing the mRNA levels of regulating proteins (AKT1, CCNB1 and CCND1) (Additional file 2: Fig. S1). Flow cytometry analysis revealed that overexpression of TRAF3IP2-AS1 remarkably induced apoptosis rate in *NONO-TFE3* tRCC cells (Fig. 2i), and opposite results were observed in 786-O cells with lowering expression of TRAF3IP2-AS1, which was confirmed by testing mRNA level of anti-apoptotic proteins BCL2 and baculoviral IAP repeat containing 5 (BIRC5) (Additional file 2: Fig. S1). Moreover, Transwell assays revealed that ectopic expression of TRAF3IP2-AS1 decreased the migration and invasion capacity of UOK109 cells, and vice versa (Fig. 2j). Similarly, matrix metalloproteinase 2 (MMP2) and MMP9 mRNA were reduced after TRAF3IP2-AS1 overexpression but increased after silencing TRAF3IP2-AS1 (Additional file 2: Fig. S1). These findings indicate that TRAF3IP2-AS1 might act as a tumor suppressor lncRNA in *NONO-TFE3* tRCC.

### NONO-TFE3 inhibits lncRNA TRAF3IP2-AS1 transcription

**Fig. 3:**
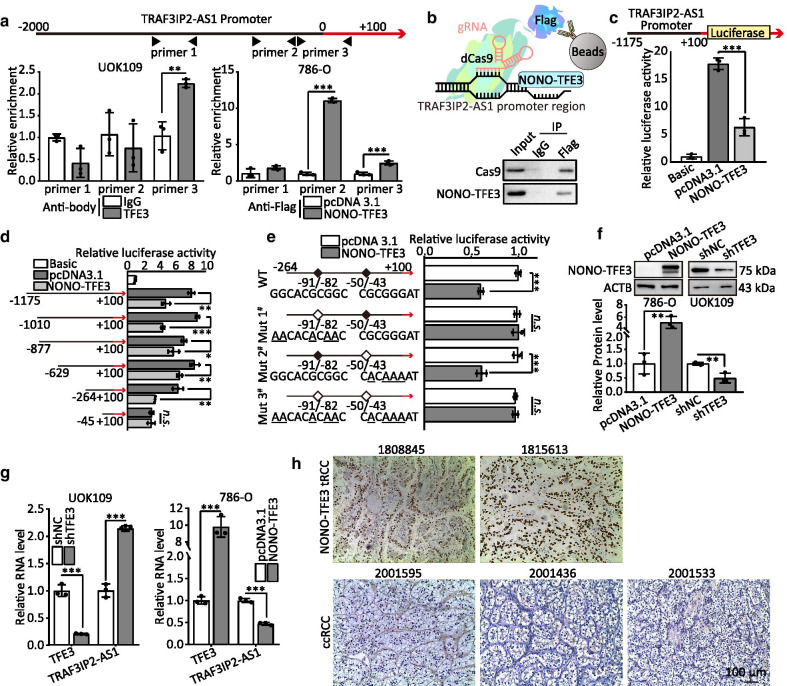
NONO-TFE3 inhibits lncRNA TRAF3IP2-AS1 transcription. **a** ChIP assays showed endogenous NONO-TFE3 binding to the TRAF3IP2-AS1 gene promoter. 786-O cells were transfected with NONO-TFE3-Flag-overexpressing vector for 48 h. The binding of NONO-TFE3 at the TRAF3IP2-AS1 promoter region was detected by a ChIP assay. **b** Schematic summary of the dCas9-gRNA-guided ChIP (upper); Western blot was performed after dCas9-gRNA-guided ChIP (lower). **c–d** HEK293T cells were co-transfected with TRAF3IP2-AS1 promoter–luciferase truncations and NONO-TFE3 plasmids, and the luciferase activity was determined using a Dual Luciferase Reporter Assay after 48 h. **e** Dual luciferase assay of HEK293T cells co-transfected with firefly luciferase constructs containing the wild-type or mutant NONO-TFE3 potential binding sites of TRAF3IP2-AS1 promoter and NONO-TFE3 plasmids were performed. **g** The protein and mRNA levels of NONO-TFE3 and the TRAF3IP2-AS1 expression levels were detected after transfection with NONO-TFE3 plasmids or shTFE3 for 48 h. **h** NONO-TFE3 immunohistochemistry was performed in paraffin sections of samples from patients. The data are presented as the mean ± SD, *P < 0.05, **P < 0.01, ***P < 0.001

To prove the hypothesis that *TRAF3IP2-AS1* is a potential target gene of NONO-TFE3 according to ChIP-seq, we conducted ChIP assays and found that NONO-TFE3 directly interacted with the NONO-TFE3 binding sites within the *TRAF3IP2-AS1* promoter in UOK109 and 786-O cells (Fig. 3a). This finding was confirmed by the result of dCas9-gRNA-guided ChIP (Fig. 3b). Furthermore, dual-luciferase reporter gene assay revealed that NONO-TFE3 fusion directly targeted the promoter of *TRAF3IP2-AS1* to negatively regulate the luciferase activity (Fig. 3c). To clarify the upstream regulatory mechanism, six promoter regions, designated as pGL3-TRAF3IP2-AS1-P1 (− 1175 ~  + 100), pGL3-TRAF3IP2-AS1-P2 (− 1010 ~  + 100), pGL3-TRAF3IP2-AS1-P3 (− 877 ~  + 100), pGL3-TRAF3IP2-AS1-P4 (− 629 ~  + 100), pGL3-TRAF3IP2-AS1-P5 (− 264 ~  + 100) and pGL3-TRAF3IP2-AS1-P6 (− 45 ~  + 100), were cloned into a luciferase reporter plasmid to identify the binding sites of NONO-TFE3 fusion. Dual-luciferase reporter assays revealed that NONO-TFE3 fusion could bind to the region of − 45 ~  + 100 (Fig. 3D). Then, to further determine the exact binding sites, this promoter region of TRAF3IP2-AS1 was cloned into a luciferase reporter plasmid and mutations were made at 3 putative binding sites, respectively. HEK293T cells were co-transfected with the pcDNA3.1-NONO-TFE3/pcDNA3.1 and Luc-WT, Luc-Mut1#, Luc-Mut2# or Luc-Mut3#. The luciferase activity of both Luc-Mut1# and Luc-Mutc3# showed no significant change, whereas Luc-Mut2# showed significantly change, indicating that the actual site of NONO-TFE3 binding to TRAF3IP2-AS1 was − 91 ~ − 82 (Fig. 3e).

Next, we altered NONO-TFE3 protein levels by loss- and gain-of-functions in vitro and detected the expression of TRAF3IP2-AS1 in UOK109 and 786-O cells, respectively. The expression of TRAF3IP2-AS1 was upregulated by knockdown of NONO-TFE3 compared with the negative control in UOK109 cells, and similar results are shown in 786-O cells (Fig. 3g). The negative correlation between NONO-TFE3 and TRAF3IP2-AS1 was confirmed using immunofluorescence (Fig. 3h). These data demonstrate that NONO-TFE3 fusion can bind to TRAF3IP2-AS1 and negatively regulate the expression of TRAF3IP2-AS1.

As NONO-TFE3 fusion has been described to play a role in the regulation of transcription of TRAF3IP2-AS1, to verify that NONO-TFE3 could promote tumor growth through decreasing TRAF3IP2-AS1, a series of rescue experiments were performed. Cell proliferation, colony formation and tumor sphere formation were inhibited by silencing the expression of NONO-TFE3 compared with the negative control in UOK109 cells, but the knockdown of TRAF3IP2-AS1 reversed this phenomenon (Additional file 2: Fig. S2A–D). Concordantly, overexpression of NONO-TFE3 significantly increased cell proliferation, colony formation and tumor sphere formation in 786-O cell line. After transfected with TRAF3IP2-AS1 overexpressed plasmid, the capacities of cell proliferation, colony formation and tumor sphere formation came back to control level (Additional file 2: Fig. S2A–D). The data of both EdU assays and flow cytometry analysis including cell cycle and apoptosis also confirmed the above results (Additional file 2: Fig. S2E–I).

**Fig. 4:**
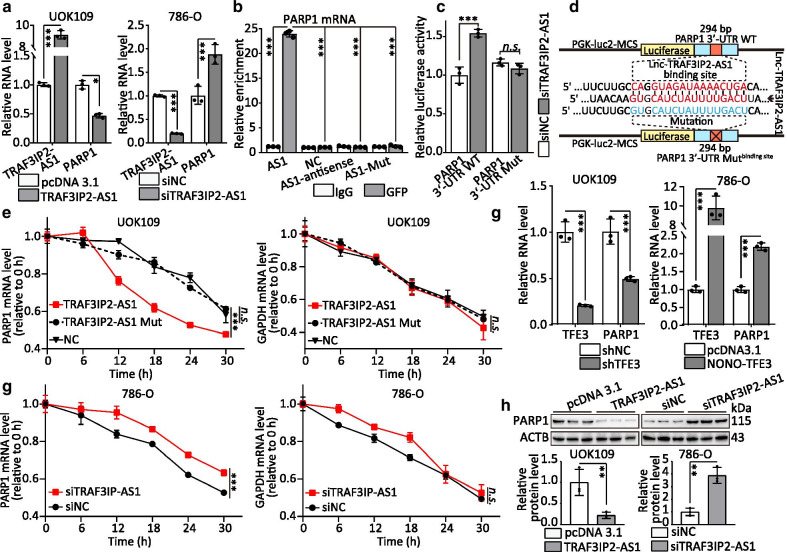
TRAF3IP2-AS1 down-regulates PARP1 mRNA by direct binding. **a** The RNA levels of TRAF3IP2-AS1 and PARP1 were detected after transfection with TRAF3IP2-AS1 plasmids or siTRAF3IP2-AS1 for 48 h. **b** Level of PARP1 mRNA detected by qRT-PCR after MS2-RIP for GFP in UOK109 cells. AS1, NC, AS1-antisense and AS1-Mut correspond to TRAF3IP2-AS1, empty vector, TRAF3IP2-AS1-antisense and TRAF3IP2-AS1 with mutation of potential binding site to PARP1 mRNA, respectively. **c–d** Dual Luciferase Reporter Assay used to detect the relative luciferase activity in HEK293 T cells co-transfected with siTRAF3IP2-AS1 and pmirGLO-PARP1 3′-UTR WT/MUT. **e** The stability of PARP1 mRNA and GAPDH mRNA in UOK109 cells transfected with TRAF3IP2-AS1 or TRAF3IP2-AS1 contained binding site mutation was measured by qRT-PCR relative to 0 h after blocking new RNA synthesis with α-amanitin and normalized to 18 s rRNA. **f** The RNA levels of NONO-TFE3 and PARP1 were detected after transfection with NONO-TFE3 plasmids or shTFE3 for 48 h. **g** The stability of PARP1 mRNA and GAPDH mRNA in 786-O cells transfected with siTRAF3IP2-AS1 was measured by qRT-PCR after treatment with α-amanitin. **h** The protein level of PARP1 was detected after transfection with TRAF3IP2-AS1 plasmids or siTRAF3IP2-AS1

Moreover, ectopic expression of NONO-TFE3 increased the migration and invasion capacity of 786-O cells, and vice versa (Fig. 4j). Interestingly, overexpression of TRAF3IP2-AS1 could reverse the high capacity of migration and invasion in 786-O cells caused by ectopic expression of NONO-TFE3. Correspondingly, mRNA levels of AKT1, CCNB1, CCND1, MMP2, MMP9, BCL2 and BIRC5 were tested to validate the above results (Additional file 2: Fig. S3). These findings reveal that NONO-TFE3 promotes *NONO-TFE3* tRCC progression through down-regulating expression of TRAF3IP2-AS1.

### TRAF3IP2-AS1 down-regulates PARP1 mRNA by direct binding

To further explore the molecular mechanism about how lncRNA TRAF3IP2-AS1 contribute to the progression phenotype of *NONO-TFE3* tRCC cells, we first analyzed the RNA sequences of TRAF3IP2-AS1 using the Encyclopedia of RNA Interactomes (ENCORI) (http://starbase.sysu.edu.cn/) [28]. Surprisingly, we identified a highly complementary region between TRAF3IP2-AS1 and PARP1 mRNA. PARP1, a well-known cancer-promoting factor, is highly expressed in multiple tumors, which induces poor prognosis of patients [29]. Upon overexpressed, TRAF3IP2-AS1 suppressed PARP1 expression in UOK109 cells, and vice versa (Fig. 4a). We analyzed the data from clinical sample and TCGA database, and the results indicated that the PARP1 expression inversely correlated with TRAF3IP2-AS1 expression (Additional file 2: Fig. S4). To validate the direct interaction of TRAF3IP2-AS1 with PARP1 mRNA, we mutated the binding site in TRAF3IP2-AS1 and performed RIP assay. The results revealed that PARP1 mRNA were significantly enriched in TRAF3IP2-AS1 (AS1) compared with TRAF3IP2-AS1-Mut (AS1-Mut) and TRAF3IP2-AS1 antisense control (AS1-antisense; Fig. 4b). The specific association between TRAF3IP2-AS1 and PARP1 mRNA was further validated by luciferase assay (Fig. 4c–d). These findings indicate that TRAF3IP2-AS1 might interact with PARP1 mRNA.

Next, to test whether TRAF3IP2-AS1 regulates the stability of PARP1 mRNA, we treated UOK109 and 786-O cells with α-amanitin to block RNA polymerase II-mediated new RNA synthesis and then measured the loss of PARP1 and GAPDH over a 24-h period. Ectopic overexpression of TRAF3IP2-AS1, but not that of TRAF3IP2-AS1 with mutation, reduced the half-life of PARP1 mRNA, whereas knockdown of TRAF3IP2-AS1 clearly elongated the half-life of PARP1 mRNA (Fig. 4e, g). Aside from this, the changes of NONO-TFE3 expression induced the corresponding alterations of PARP1 mRNA expression (Fig. 4f), and the protein level of PARP1 decreased by ectopic expression of TRAF3IP2-AS1 in UOK109 cell line (Fig. 4h). Taken together, lncRNA TRAF3IP2-AS1 could bind to PARP1 mRNA and reduce the half-life of PARP1 mRNA.

Since TRAF3IP2-AS1 can regulate the expression of PARP1 mRNA in UOK109, rescue experiments were performed to confirm the hypothesis of TRAF3IP2-AS1 mediating *NONO-TFE3* tRCC progression through PARP1. Our results showed that overexpression of TRAF3IP2-AS1 inhibited the proliferation, colony and sphere formation of UOK109 cells, which could be markedly prevented by transfected with PARP1 overexpressed plasmid (Additional file 2: Fig. S5A–C). Moreover, the results of EdU assay and flow cytometry indicated that upregulation of PARP1 expression reversed the inhibition of cell proliferating activity caused by overexpression of TRAF3IP2-AS1, and vice versa (Additional file 2: Fig. S5D–G). Flow cytometry analysis revealed that TRAF3IP2-AS1 remarkably increased apoptosis of UOK109 cells, which was fully reversed to control levels by overexpression of PARP1 (Additional file 2: Fig. S5H–I). Ectopic expression of TRAF3IP2-AS1 inhibited the migration and invasion capacity of UOK109 cells, and vice versa (Additional file 2: Fig. S5J–K). Interestingly, overexpression of PARP1 reversed the low capacity of migration and invasion in UOK109 cells caused by ectopic expression of TRAF3IP2-AS1. Correspondingly, AKT1, CCNB1, CCND1, MMP2, MMP9, BCL2 and BIRC5 mRNA levels were changed accordingly with down-/up-regulation of PARP1 and TRAF3IP2-AS1 (Additional file 2: Fig. S6). Taken together, the data confirm that TRAF3IP2-AS1 can mediate *NONO-TFE3* tRCC progression through regulating the expression PARP1.

### TRAF3IP2-AS1 accelerates the decay of PARP1 mRNA by recruitment of m^6^A methyltransferase complex

**Fig. 5:**
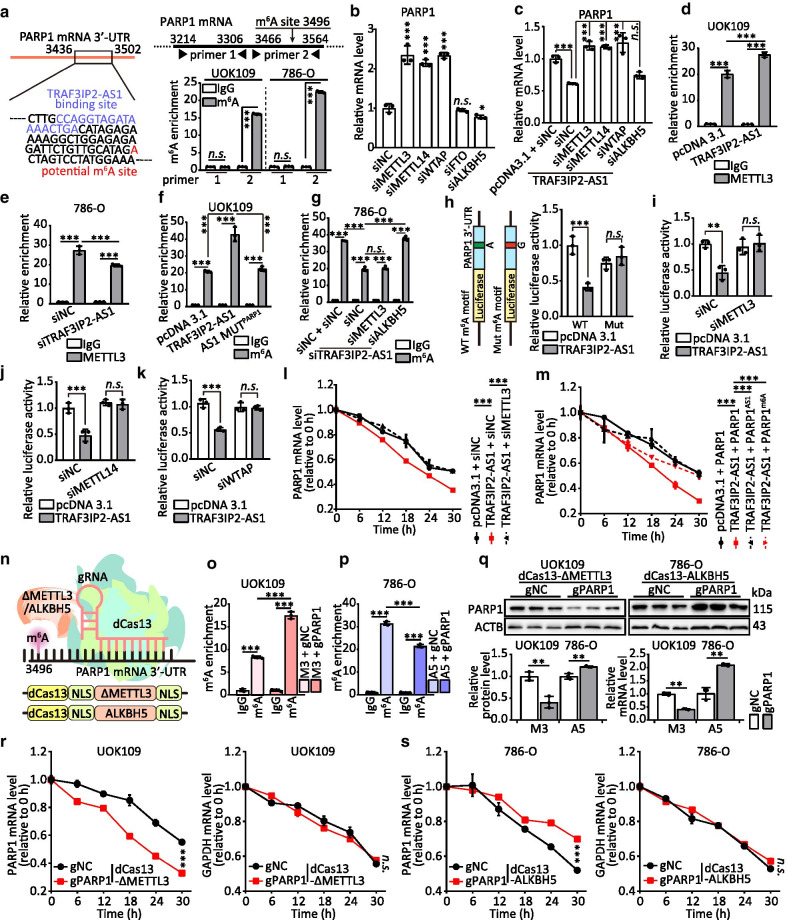
TRAF3IP2-AS1 accelerates the decay of PARP1 mRNA by recruitment of m^6^A methyltransferase complex. **a** The location of TRAF3IP2-AS1 binding site and a potential m^6^A site are indicated. Abundance of PARP1 transcript among mRNA immunoprecipitated with anti-m^6^A antibody was measured by qRT-PCR and normalized to IgG. **b–c** The mRNA level of PARP1 was detected by qRT-PCR after transfected with indicated vectors or siRNAs. **d–e** METTL3 was immunoprecipitated followed by qRT-PCR for assessing the association of the indicated PARP1 mRNA with METTL3 after overexpression or knockdown of TRAF3IP2-AS1. **f–g** Abundance of PARP1 among mRNA immunoprecipitated with anti-m^6^A antibody from cells transfected with indicated vectors or siRNA/ASO was measured by qRT-PCR. **h** PARP1-3′-UTR of the wild-type or containing a m^6^A consensus sequence mutant (A to G) was fused with a luciferase reporter. Luciferase activity of PARP1-3′-UTR was measured and normalized to Renilla luciferase activity. **p–k** Luciferase activity of PARP1-3′-UTR was measured after co-transfected with TRAF3IP3-AS1 and siMETTL3/siMEETTL14/siWTAP. **l–m** The stability of PARP1 mRNA in cells co-transfected with indicated vectors or siRNAs after treatment with α-amanitin. **n** Schematic illustration of targeted RNA methylation system. **o–p** Abundance of PARP1 among mRNA immunoprecipitated with anti-m^6^A antibody from cells transfected with indicated gRNA was measured by qRT-PCR. **q** The protein and mRNA level of PARP1 were measured by qRT-PCR and Western blot after transfected with indicated gRNA. **r–s** The stability of PARP1 and GAPDH mRNA in cells transfected with indicated gRNA after treatment with α-amanitin. The data are presented as the mean ± SD, *P < 0.05, **P < 0.01, ***P < 0.001

Furtherly, according to the analysis of PARP1 mRNA from WHISTLE (https://whistle-epitranscriptome.com/) [30], we found that an *N*^6^-methyladenosie (m^6^A) modification site is near the binding site of TRAF3IP2-AS1 in PARP1 mRNA (Fig. 5a). Therefore, to explore the potential mechanism by which TRAF3IP2-AS1 reduces the stability of PARP1 mRNA, we wondered whether m^6^A modification plays a role in the decay of PARP1 mRNA. The results of m^6^A RIP analyses showed that m^6^A was significantly enriched at the prediction site (Fig. 5a). To figure out which proteins play an important role in decay of PARP1 mRNA, siRNAs of RNA methylases including methyltransferase like 3 (METTL3), METTL14 or Wilms tumor 1-associating protein (WTAP) and RNA demethylases alkB homolog 5 (ALKBH5) or fat mass and obesity associated (FTO) were designed and transfected into UOK109 individually. The PARP1 mRNA level was markedly increased after METTL3, METTL14 and WTAP knockdown using RNA interference, but knockdown of ALKBH5 or FTO did not affect the expression of PARP1 mRNA (Fig. 5b; Additional file 2: S7A). Moreover, knockdown of METTL3, METTL14 and WTAP abolished the impact on the mRNA level of PARP1 upon TRAF3IP2-AS1 ectopic expression (Fig. 5c; Additional file 2: S7C-D). Interestingly, there were no changes in the levels of m^6^A-related proteins in TRAF3IP2-AS1-overexpressing cells (Additional file 2: Fig. S7B). In addition, METTL3 was remarkably enriched in PARP1 mRNA in TRAF3IP2-AS1 overexpression group compared with that in empty vector group, and a reduction in the enrichment of METTL3 on PARP1 mRNA was observed following knockdown of TRAF3IP2-AS1 (Fig. 5d–e). The mutation of binding site on TRAF3IP2-AS1 could abolish the increased enrichment of m^6^A in PARP1 mRNA (Fig. 5f). Interestingly, the decreased enrichment of m^6^A caused by silence of TRAF3IP2-AS1 was basically unchanged after METTL3 knockdown (Fig. 5g).

Furthermore, the luciferase reporter assay revealed that luciferase activity of PARP1-3′-UTR could be downregulated by overexpression of TRAF3IP2-AS1, but TRAF3IP2-AS1 failed to decrease the luciferase activity of the reporter construct bearing mutant PARP1-3′-UTR (Fig. 5h). Likewise, the decreased luciferase activity caused by TRAF3IP2-AS1 overexpression was abolished by METTL3, METTL14 or WTAP deficiency (Fig. 5i–k). The decreased decay rates of PARP1 mRNA were nearly restored back to normal by knockdown of METTL3 in UOK109 cells (Fig. 5l; Additional file 2: Fig. S7E). Consistent with the findings reported above, the decay rates of PARP1 mRNA with mutation of TRAF3IP2-AS1 binding site (PARP1^AS1^) or m^6^A modification site (PARP1^m6A^) showed no response to overexpression of TRAF3IP2-AS1 (Fig. 5m; Additional file 2: Fig. S7F). These findings indicate that TRAF3IP2-AS1 could interact with PARP1 mRNA and reduce its stability by stimulating m^6^A mRNA modification.

Targeted RNA methylation system was applied to confirm that the m^6^A modifications of PARP1 mRNA could play a critical role in *NONO-TFE3* tRCC tumor progression. Tethering catalytically inactivated Cas13 (dCas13) to m^6^A writer/eraser/readers could allow programmable installation of m^6^A at sites specified by a Cas13 guide RNA (gRNA) [24, 25]. METTL3^273–580^ (ΔMETTL3/M3) increased the enrichment of m^6^A in PARP1 mRNA in UOK109 cells, and full length ALKBH5 (A5) decreased the enrichment of m^6^A in 786-O cells (Fig. 5n–p). Compared with negative control (gNC), the protein and mRNA level of PARP1 were decreased in UOK109 cells transfected with dCas13-ΔMETTL3 and gRNA targeting PARP1 mRNA (gPARP1), and the protein and mRNA level of PARP1 were increased in 786-O cells transfected with dCas13-ALKBH5 and gPARP1 (Fig. 5q). Correspondingly, the elevated m^6^A modification of PARP1 mRNA accelerated the mRNA decay (Fig. 5r), and the decreased m^6^A modification of PARP1 mRNA kept the mRNA stability (Fig. 5s). Collectively, TRAF3IP2-AS1 could recruit m^6^A methyltransferase complex to PARP1 mRNA to elevate m^6^A modification, which might reduce half-life of PARP1 mRNA.

To further explore the biological function of m^6^A modification of PARP1 mRNA in *NONO-TFE3* tRCC, CCK-8, colony formation, tumor sphere formation, EdU, flow cytometry and Transwell assays were performed. The results revealed that cell proliferation (Additional file 2: Fig. S8A–D), cell cycle (Additional file 2: Fig. S8E), migration and invasion (Additional file 2: Fig. S8G) were inhibited by the elevated m^6^A modification of PARP1 mRNA compared with the negative control in UOK109 cells, and ALKBH5-gPARP1 enhanced these behaviors of 786-O cells. Flow cytometric experiments showed that up-regulation of m^6^A in PARP1 mRNA obviously increased the apoptosis of UOK109 cells, and vice versa (Additional file 2: Fig. S8F). Correspondingly, mRNA levels of AKT1, CCNB1, CCND1, MMP2, MMP9, BCL2 and BIRC5 were tested to validate the above results (Additional file 2: Fig. S8H-I). Overall, TRAF3IP2-AS1 could modulate the progression of *NONO-TFE3* tRCC by elevating m^6^A modification of PARP1 mRNA.

### YTHDF2 mediates the decay of PARP1 mRNA to regulate *NONO-TFE3* tRCC progression

**Fig. 6:**
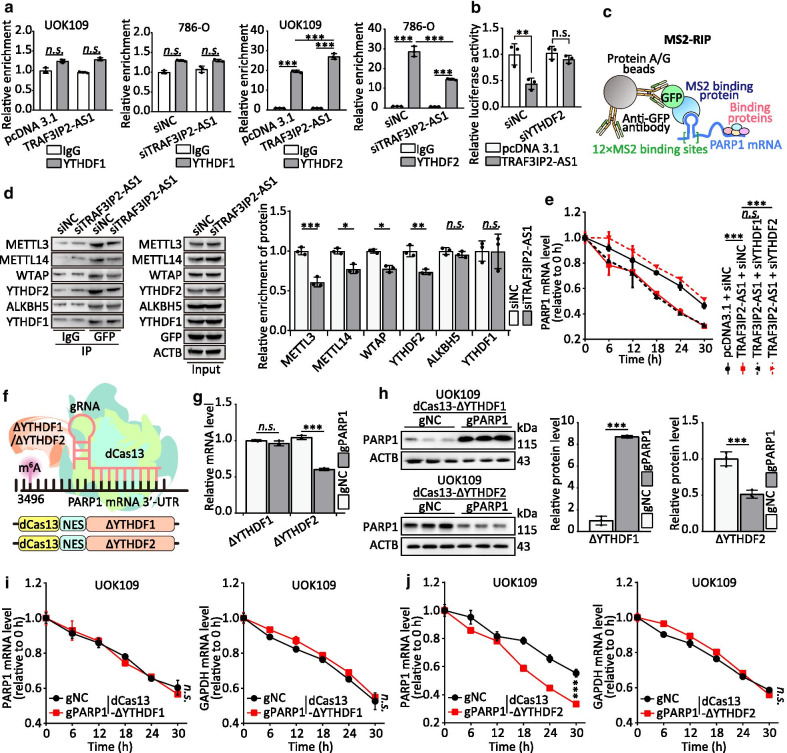
YTHDF2 mediates the decay of PARP1 mRNA to regulate *NONO-TFE3* tRCC progression. **a** YTHDF1/2 was immunoprecipitated followed by qRT-PCR for assessing the association of PARP1 mRNA with YTHDF1/2 after overexpression or knockdown of TRAF3IP2-AS1. **b** Luciferase activity of PARP1-3′-UTR was measured after co-transfected with TRAF3IP3-AS1 and siYTHDF2. **c** Schematic illustration of MS2-RIP. **d** Abundance of m^6^A relative proteins among MS2-RIP with anti-GFP antibody from cells transfected with indicated plasmid was measured by Western blot. **e** The stability of PARP1 mRNA in cells co-transfected with indicated vectors or siRNAs after treatment with α-amanitin. **f** Schematic illustration of targeted RNA methylation system. **g–h** The mRNA (**g**) and protein (**h**) level of PARP1 were measured by qRT-PCR and Western blot after transfected with indicated gRNA. **i–j** The stability of PARP1 and GAPDH mRNA in cells transfected with indicated gRNA after treatment with α-amanitin. The data are presented as the mean ± SD, *P < 0.05, **P < 0.01, ***P < 0.001

Because TRAF3IP2-AS1 and m^6^A methylation appeared to reduce the expression of PARP1, we speculated that PARP1 transcript was a target of YTHDF2 (YTH N6-methyladenosine RNA binding protein 2). As assessed by RIP analyses, YTHDF2 interacted strongly with PARP1, not YTHDF1 (Fig. 6a), and the same phenomenon was observed in the luciferase reporter assay (Fig. 6b). These results were also confirmed by MS2-RIP/western blot analysis in UOK109 cells (Fig. 6c–d). Consistent with the findings reported above, the decreased decay rates of PARP1 mRNA were nearly restored back to normal by knockdown of YTHDF2 in UOK109 cells (Fig. 5e; Additional file 2: S9A). Targeted m^6^A read system was applied to confirm the above results [23]. YTHDF2^1–400^ (ΔYTHDF2) decreased the protein and mRNA level of PARP1 in UOK109 cells transfected with gPARP1 and YTHDF1^1–350^ (ΔYTHDF1) increased the protein level of PARP1 (Fig. 6f–h). Consistent with prediction, ΔYTHDF1 showed no significant impact in the mRNA level of PARP1 in UOK109 cells transfected with gPARP1. Correspondingly, ΔYTHDF2, but not ΔYTHDF1, and gPARP1 accelerated the mRNA decay of PARP1 (Fig. 6i–j). Collectively, YTHDF2 could induce PARP1 mRNA degradation.

CCK-8, colony formation, tumor sphere formation, EdU, flow cytometry and Transwell assays were performed to illuminate the impact of YTHDF2 to the behavior of UOK109 cells. The results revealed that cell proliferation (Additional file 2: Fig. S9B–E), cell cycle (Additional file 2: Fig. S9F), migration and invasion (Additional file 2: Fig. S9H) were inhibited by transfection with ΔYTHDF2 and gPARP1 compared with the negative control in UOK109 cells. Flow cytometric experiments showed that ΔYTHDF2 and gPARP1 obviously increased the apoptosis of UOK109 cells (Additional file 2: Fig. S9G). Correspondingly, mRNA levels of AKT1, CCNB1, CCND1, MMP2, MMP9, BCL2 and BIRC5 were tested to validate the above results (Additional file 2: Fig. S9I–J). Taken together, these results indicate that YTHDF2 mediates *NONO-TFE3* tRCC progression by recognizing PARP1 mRNA and facilitating the mRNA decay.

### TRAF3IP2-AS1 functions as a ceRNA to sponge miRNAs

**Fig. 7:**
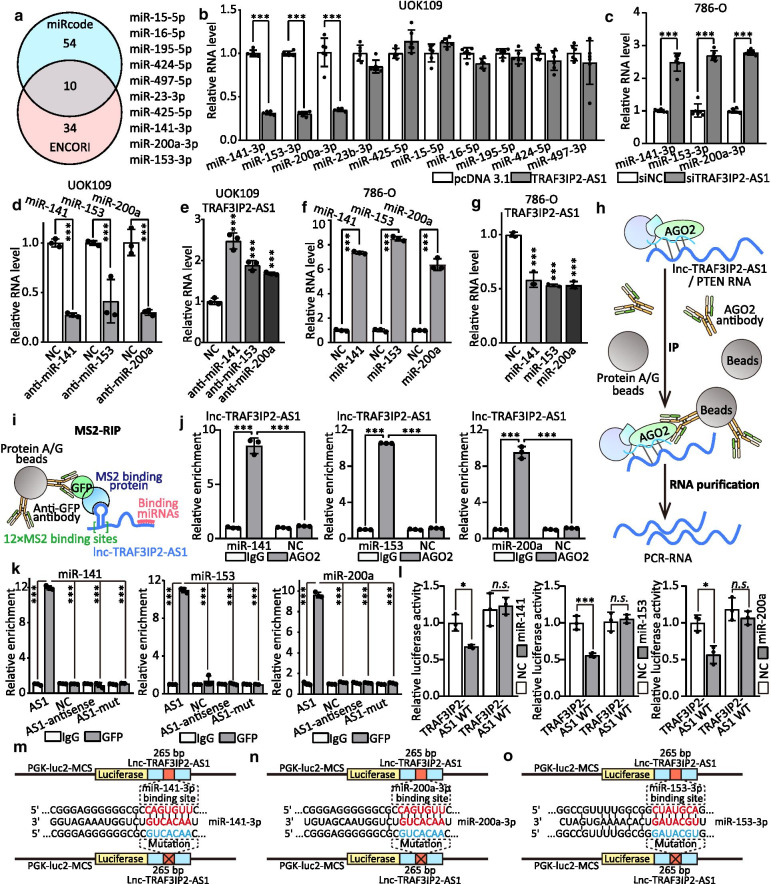
TRAF3IP2-AS1 functions as a ceRNA and sponges miRNAs. **a** Schematic of the selection for the direct downstream target of TRAF3IP2-AS1. **b–c** The effect of TRAF3IP2-AS1 on multiple miRNAs expression in UOK109 cells was analyzed by qRT-PCR after overexpression or knockdown of TRAF3IP2-AS1. **d–f** The RNA levels of multiple miRNAs and TRAF3IP2-AS1 were analyzed via qRT-PCR in UOK109 cells after transfected with miRNA inhibitor, respectively. **e–g** The RNA levels of multiple miRNAs and TRAF3IP2-AS1 were analyzed via qRT-PCR in 786-O cells after overexpression miRNAs, respectively. **h–i** Model of AGO2-RIP/MS2-RIP assay. **j** RIP assays were performed using AGO2 antibody in UOK109 cells, and then, the enrichment ofTRAF3IP2-AS1was detected by qRT-PCR. **k** MS2-RIP-derived RNA was examined by qRT-PCR. The levels of the qRT-PCR products were normalized relative to IgG control. **l** HEK293T cells were co-transfected with miRNA mimics, respectively, and wild-type or mutant TRAF3IP2-AS1 luciferase reporter vector, and luciferase reporter activity was detected. **m–o** Schematic illustration of TRAF3IP2-AS1 wild type and mutation luciferase reporter vectors. The data are presented as the mean ± SD, *P < 0.05, **P < 0.01, ***P < 0.001

Previous studies have indicated that lncRNAs exert critical roles in regulating gene expression by serving as a sponge for miRNAs [31]. The intracellular distribution of TRAF3IP2-AS1 suggested that TRAF3IP2-AS1 might also have a post-transcriptional regulation function that contributes to *NONO-TFE3* tRCC progression. Then, we predicted potential miRNA targeting sites on TRAF3IP2-AS1 using ENCORI and miRcode (http://www.mircode.org) database and screened out ten candidate miRNAs (Fig. 7a). Then, the validation of qRT-PCR showed that the expression of miR-200a-3p, miR-153-3p and miR-141-3p were reduced in UOK109 cells upon TRAF3IP2-AS1 overexpression (Fig. 7b). Similarly, silencing the TRAF3IP2-AS1 expression increased the level of miR-200a-3p, miR-153-3p and miR-141-3p in 786-O cells (Fig. 7c). Interestingly, the expression of TRAF3IP2-AS1 was changed accordingly with down-/up-regulation of miR-200a-3p, miR-153-3p or miR-141-3p (Fig. 7d–g). In addition, we confirmed, by down-/up-regulation of NONO-TFE3, that NONO-TFE3 had potential indirect effects on these miRNAs expression (Additional file 2: Fig. S10).

To determine the interaction between TRAF3IP2-AS1 and the listed miRNAs, we performed RIP assays and found that TRAF3IP2-AS1 could directly interact with AGO2, which has been identified as a critical member of RNA-induced silencing complex (RISC) and closely correlates with mRNA repression mediated by miRNA, while miR-200a-3p, miR-153-3p and miR-141-3p were up-regulated (Fig. 7h, j). Moreover, the results of MS2-RIP revealed that miR-200a-3p, miR-153-3p and miR-141-3p was significantly enriched in TRAF3IP2-AS1 (AS1) compared with TRAF3IP2-AS1-Mut (AS1-Mut) and TRAF3IP2-AS1 antisense control (AS1-antisense; Fig. 7i, k). Dual-luciferase assay was processed to confirm the interaction between miRNAs and TRAF3IP2-AS1. As shown in Fig. 7l–o, luciferase activities were repressed by co-transfection with miR-200a-3p, miR-153-3p or miR-141-3p compared with the control. However, this inhibitory effect was abolished by mutation of the putative miRNAs binding site in the TRAF3IP2-AS1. Taken together, these results indicate that miR-200a-3p, miR-153-3p and miR-141-3p can bind to the predicted sites in TRAF3IP2-AS1 in UOK109 cells.

To further illuminate the biological function of miRNAs in *NONO-TFE3* tRCC, miR-200a-3p, miR-153-3p and miR-141-3p plasmid or inhibitor was used to upregulate or downregulate miRNAs expression in UOK109 and 786-O cells. CCK-8 assays showed that repression of miR-200a-3p, miR-153-3p and miR-141-3p obviously inhibited proliferation of UOK109 cells, while the overexpression of miRNAs led to inverse effects in 786-O cells (Additional file 2: Fig. S11A). Furthermore, the results of colony formation (Additional file 2, Fig. S11B-D), tumor sphere formation (Additional file 2: Fig. S11E-F), EdU (Additional file 2: Fig. S11G-H), flow cytometry (Additional file 2: Fig. S12A) and Transwell assays (Additional file 2: Fig. S12C) revealed that cell proliferation, cell cycle, migration and invasion were enhanced by overexpression of miR-200a-3p, miR-153-3p and miR-141-3p compared with the negative control in UOK109 cells. Flow cytometric experiments showed that increased miRNAs expression obviously inhibited apoptosis in UOK109 cells, and vice versa (Additional file 2: Fig. S12B). Correspondingly, the mRNA levels of AKT1, CCNB1, CCND1, MMP2, MMP9, BCL2 and BIRC5 were tested to validate the above results (Additional file 2: Fig. S13). Collectively, the results reveal that TRAF3IP2-AS1 functions as a ceRNA to sponge miR-200a-3p, miR-153-3p and miR-141-3p.

### TRAF3IP2-AS1 modulates the expression of PTEN through post-transcriptional regulation of miRNAs

**Fig. 8:**
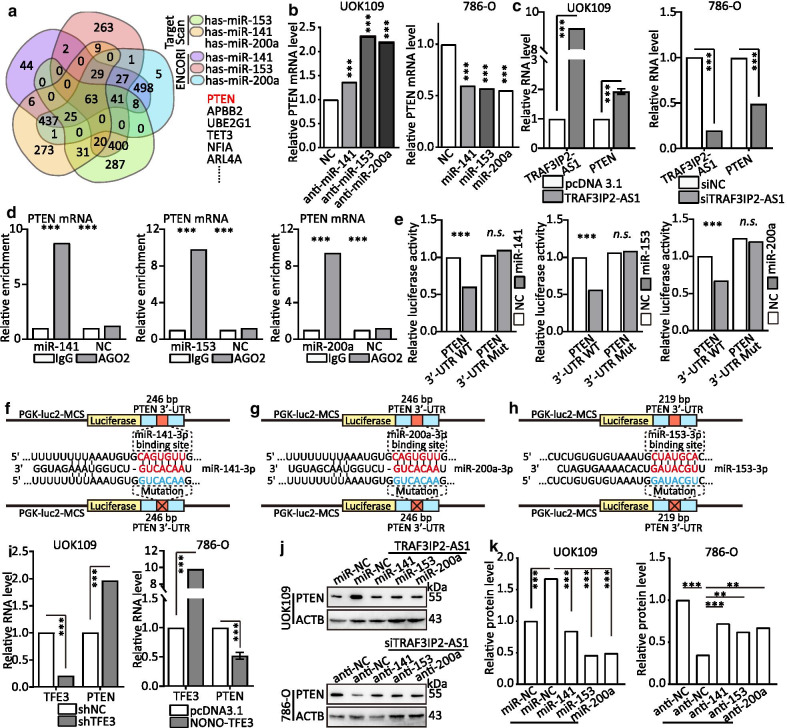
TRAF3IP2-AS1 modulates the expression of PTEN through post-transcriptional regulation of miRNAs. **a** Schematic of the selection for the direct downstream target of TRAF3IP2-AS1. **b–c** The effect of multiple miRNAs expression on PTEN mRNA in UOK109 cells was analyzed by qRT-PCR after overexpression or knockdown of miRNAs, respectively. **d** RIP assays were performed using AGO2 antibody in UOK109 cells, and then, the enrichment of PTEN mRNA was detected by qRT-PCR. **e** HEK293T cells were co-transfected with miRNA mimics, respectively, and wild-type or mutant PTEN 3′-UTR luciferase reporter vector, and luciferase reporter activity was detected. **f–h** Schematic illustration of PTEN 3′-UTR wild type and mutation luciferase reporter vectors. **i** The mRNA levels of NONO-TFE3 and PTEN were detected after transfection with NONO-TFE3 plasmids or shTFE3 for 48 h. **j–k** The protein level of PTEN was detected after co-transfection with indicated vectors, siRNAs/ASOs or miRNAs inhibitor for 48 h. The data are presented as the mean ± SD, **P < 0.01, ***P < 0.001

Interestingly, through ENCORI and TargetScan (http://www.targetscan.org/) analysis, PTEN was found to be one of the most commonly potential target genes of miR-200a-3p, miR-153-3p and miR-141-3p (Fig. 8a; Additional file 2: S14A). Overexpression or downregulation of miR-200a-3p, miR-153-3p and miR-141-3p caused a significant decrease or increase in PTEN expression at mRNA and protein levels (Fig. 8b; Additional file 2: S14B–D). In addition, the mRNA level of PTEN was significantly increased or decreased with TRAF3IP2-AS1 knockdown or upregulation (Fig. 8c). We analyzed the data from clinical sample and TCGA database, and the results indicated that the expression of PTEN is positively correlated with median the level of TRAF3IP2-AS1 (Additional file 2: Fig. S15). According to these findings, we hypothesized that TRAF3IP2-AS1, these miRNAs and PTEN might form a common ceRNA network in *NONO-TFE3* tRCC.

To test our hypothesis, we performed RIP assays and found that the ectopic expression of miR-200a-3p, miR-153-3p or miR-141-3p augmented the enrichment of AGO2 at PTEN mRNA (Fig. 8d). Indeed, luciferase reporter assays showed that WT-PTEN-driven luciferase expression was significantly inhibited by co-transfection with the miR-200a-3p, miR-153-3p or miR-141-3p compared with the control. However, this inhibitory effect was abolished by mutation of the putative miRNAs binding site in the PTEN 3′- UTR (Fig. 8e). Correspondingly, down-/up-regulation of NONO-TFE3 could change the mRNA level of PTEN accordingly (Fig. 8i). Additionally, we performed rescue assays to evaluate whether TRAF3IP2-AS1 regulates PTEN by competing for miR-200a-3p, miR-153-3p and miR-141-3p. As shown in Fig. 8j–k, the results showed that overexpression of TRAF3IP2-AS1 increased PTEN levels and ectopic expression of miR-200a-3p, miR-153-3p or miR-141-3p repressed this increase, whereas suppression of TRAF3IP2-AS1 decreased PTEN levels and effects of miRNAs inhibitor impaired this downregulation. Taken together, these results indicate that miR-200a-3p, miR-153-3p and miR-141-3p can bind to the predicted sites in the 3′-UTR of PTEN mRNA and mediated PTEN expression levels in *NONO-TFE3* tRCC.

To further explore the biological function of miRNAs and PTEN in *NONO-TFE3* tRCC, CCK-8, colony formation, tumor sphere formation, EdU, flow cytometry and Transwell assays were performed. The results revealed that cell proliferation (Additional file 2: Fig. S16), cell cycle (Additional file 2: Fig. S17A, C), migration and invasion (Additional file 2: Fig. S17E) were enhanced by overexpression of miR-200a-3p, miR-153-3p and miR-141-3p compared with the negative control in UOK109 cells, and ectopic expression of PTEN repressed this increase. Flow cytometric experiments showed that down-regulation of PTEN obviously prevented the apoptosis increased by miRNAs inhibitor in UOK109 cells, and vice versa (Additional file 2: Fig. S17B, D). Correspondingly, mRNA levels of AKT1, CCNB1, CCND1, MMP2, MMP9, BCL2 and BIRC5 were tested to validate the above results (Additional file 2: Fig. S18). These findings illuminated the existence of a TRAF3IP2-AS1-miRNAs-PTEN regulatory axis. Collectively, PTEN is a target gene of miR-200a-3p, miR-153-3p and miR-141-3p and is indirectly regulated by TRAF3IP2-AS1.

## Discussion

**Fig. 9:**
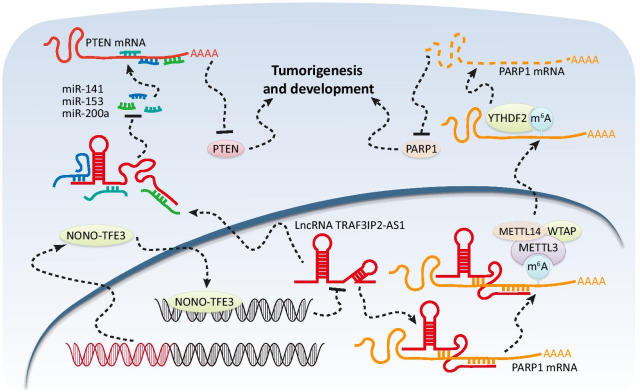
Schematic diagram for the mechanisms of TRAF3IP2-AS1 functioning as both an mRNA decay accelerator and a miRNA sponge to inhibit progression of *NONO-TFE3* tRCC

A growing body of research indicates that dysregulated lncRNAs participate in many physiological and pathological processes during cancer progression [32, 33]. Although dysregulation of certain lncRNAs in tumorigenesis is a recognized phenomenon [34, 35], the biological function and underlying molecular mechanism of most lncRNAs still remain undetermined. In this study, we clarified that lncRNA TRAF3IP2-AS1 expression was downregulated by overexpression of NONO-TFE3-fusion protein in *NONO-TFE3* tRCC. Importantly, TRAF3IP2-AS1 regulated the expression of two tumor-related specific genes, PARP1 and PTEN. Mechanistically, TRAF3IP2-AS1 directly bound to PARP1 mRNA, thereby promoted the m^6^A modification of PARP1 mRNA, resulting in decay of PARP1 mRNA. However, low expression of TRAF3IP2-AS1 caused upregulation of PARP1 by decreasing the PARP1 mRNA decay in *NONO-TFE3* tRCC. Meanwhile, sponging miR-200a-3p, miR-153-3p and miR-141-3p was the other biological function of TRAF3IP2-AS1. MiR-200a-3p, miR-153-3p and miR-141-3p could bind to the 3′-UTR of PTEN mRNA and mediated PTEN expression levels. Last, we demonstrated that TRAF3IP2-AS1 acts as a tumor suppressor during tumor progression and upregulation of TRAF3IP2-AS1 can inhibit cell proliferation, migration and invasion in *NONO-TFE3* tRCC (Fig. 9). This study elucidates a new regulation network in *NONO-TFE3* tRCC, which might provide novel insights into molecular based diagnosis and treatment of *NONO-TFE3* tRCC.

The complex and precise regulatory function of lncRNAs, such as molecular scaffold or decoy, was mediated by RNA-RNA interaction basically [36, 37]. A current study by Gu et al. showed that lncRNA LBCS interacts directly with 5′-UTR region of androgen receptor (AR) mRNA and then inhibits AR expression by forming a LBCS-AR mRNA complex, and the inhibitive effect of LBCS on AR translation was in hnRNPK dependent manner [13]. Besides this, AR expression could be enhanced by direct interaction between lncRNA-ARLNC1 and AR mRNA [15]. Here, we described that TRAF3IP-AS1 interacted with 3′-UTR of PARP1 mRNA directly and then inhibited PARP1 expression by promoting the degradation of PARP1 mRNA. We revealed that m^6^A modification of PARP1 was mediated by METTL14, and the decay of PARP1 mRNA was in YTHDF2 dependent manner.

The vital role of m^6^A RNA methylation is regulating the stability of RNA. The formation and catalysis m^6^A RNA modification is mediated by a methyltransferase complex consisting of METTL3, METTL14 and WTAP [38, 39]. The main function of METTL14, without enzymatically active, is to facilitate METTL3 binding to RNA. Diverse outcomes of m^6^A-methylation to post-transcription rely on its readers. YTHDF2 is the most effective m^6^A reader that weakens RNA stability by recognizing and distributing m^6^A-containing RNAs to processing bodies [40]. Here, it has been shown that m^6^A labeling promoted the degradation of PARP1 mRNA through YTHDF2. m^6^A was also found in lncRNAs, ribosomal RNAs (rRNAs), transfer RNAs (tRNAs) and even in circular RNAs (circRNAs), which therefore opens a new window for the pathway of RNA degradation [41]. A current study by Park et al. showed that m^6^A-containing circular RNAs are degraded by the YTHDF2 pathway [42].

CeRNA plays an important role in post-transcriptional regulation by forming extensive ceRNA network involving different kinds of RNA, such as lncRNAs, mRNAs, circRNAs and miRNAs. In this study, we found that TRAF3IP2-AS1 could function as a ceRNA and modulate the expression of PTEN through post-transcriptional regulation of miR-200a-3p, miR-153-3p and miR-141-3p. Recent studies have shown that ceRNA network plays an important role in tumor differentiation, proliferation, metastasis and other tumor development processes. For example, lncRNA UCA1 represses the host immune systems by upregulating the PD-L1 level of gastric cancer cells by reducing miRNAs expression [12]. Interestingly, α_1_ integrinI (ITGA1) mRNA and adenylyl cyclase 9 (ADCY9) mRNA competed for binding to miR-181b, and ZEB1 upregulated ITGA1 to activate a miR-181b-regulated ceRNA network that increased metastasis of lung adenocarcinomas [43].

## Conclusion

In summary, we observed that the expression of TRAF3IP2-AS1 was down-regulated by overexpressed NONO-TFE3-fusion protein in *NONO-TFE3* tRCC. As a tumor suppressor gene, upregulation of TRAF3IP2-AS1 markedly inhibited PARP1 expression by binding to its mRNA directly to stimulate m^6^A methylation which led to mRNA decay of PARP1 and enhanced the expression of PTEN through absorbing miR-200a-3p, miR-153-3p and miR-141-3p. Our findings may facilitate better understanding of *NONO-TFE3* tRCC pathogenesis and provide new insight into lncRNA-based diagnosis and treatment of *NONO-TFE3* tRCC and other human malignancies.

## Supplementary Information

**Additional file 1**. Supplementary Materials and Primers.

**Additional file 2**. Supplementary Figures.

## Data Availability

The datasets used and/or analyzed during the current study are available from the corresponding author on reasonable request.

## Abbreviations

ACTB: Actin, beta
ALKBH5: AlkB homolog 5, RNA demethylase
AGO2: Argonaute 2
CCK8: Cell Counting Kit-8
ChIP: Chromatin immunoprecipitation
CRISPR: Clustered regularly interspaced short palindromic repeats
FTO: Fat mass and obesity associated
GFP: Green fluorescent protein
m^6^A: *N*^6^-Methyladenosine
METTL14: Methyltransferase like 14
METTL3: Methyltransferase like 3
MiT/TFE: Microphthalmia family of bHLH-LZ transcription factors
NES: Nuclear export signal
NLS: Nuclear localization signal
NONO: Non-POU domain containing octamer binding
PARP1: Poly ADP-ribose polymerase
PI: Propidium iodide
PRCC: Proline rich mitotic checkpoint control factor
PTEN: Phosphatase and tensin homolog
RIP: RNA immunoprecipitation
TFE3: Transcription factor binding to IGHM enhancer 3
TRAF3IP2-AS1: TRAF3IP2 antisense RNA 1
tRCC: Translocation renal cell carcinoma
WTAP: Wilms tumor 1-associating protein
YTHDF: YTH N6-methyladenosine RNA binding protein

## References

[1] Moch H, Cubilla AL, Humphrey PA, Reuter VE (2016). Ulbright TMJEU: the 2016 WHO classification of tumours of the urinary system and male genital organs—part A: renal, penile, and testicular. Tumours.

[2] Argani P, Lui MY, Couturier J, Bouvier R, Fournet JC, Ladanyi M (2003). A novel CLTC-TFE3 gene fusion in pediatric renal adenocarcinoma with t(X;17)(p11.2;q23). Oncogene.

[3] Argani P, Zhong M, Reuter VE, Fallon JT, Epstein JI, Netto GJ, Antonescu CR (2016). TFE3-fusion variant analysis defines specific clinicopathologic associations among Xp11 translocation cancers. Am J Surg Pathol.

[4] Argani P, Antonescu CR, Couturier J, Fournet JC, Sciot R, Debiec-Rychter M, Hutchinson B, Reuter VE, Boccon-Gibod L, Timmons C (2002). PRCC-TFE3 renal carcinomas: morphologic, immunohistochemical, ultrastructural, and molecular analysis of an entity associated with the t(X;1)(p11.2;q21). Am J Surg Pathol.

[5] Clark J, Lu YJ, Sidhar SK, Parker C, Gill S, Smedley D, Hamoudi R, Linehan WM, Shipley J, Cooper CS (1997). Fusion of splicing factor genes PSF and NonO (p54nrb) to the TFE3 gene in papillary renal cell carcinoma. Oncogene.

[6] Huang W, Goldfischer M, Babayeva S, Mao Y, Volyanskyy K, Dimitrova N, Fallon JT, Zhong M (2015). Identification of a novel PARP14-TFE3 gene fusion from 10-year-old FFPE tissue by RNA-seq. Genes Chromosomes Cancer.

[7] Kauffman EC, Ricketts CJ, Rais-Bahrami S, Yang Y, Merino MJ, Bottaro DP, Srinivasan R, Linehan WM (2014). Molecular genetics and cellular features of TFE3 and TFEB fusion kidney cancers. Nat Rev Urol.

[8] Argani P, Hicks J, De Marzo AM, Albadine R, Illei PB, Ladanyi M, Reuter VE, Netto GJ (2010). Xp11 translocation renal cell carcinoma (RCC): extended immunohistochemical profile emphasizing novel RCC markers. Am J Surg Pathol.

[9] Huan C, Kelly ML, Steele R, Shapira I, Gottesman SR, Roman CA (2006). Transcription factors TFE3 and TFEB are critical for CD40 ligand expression and thymus-dependent humoral immunity. Nat Immunol.

[10] Tian G, Erman B, Ishii H, Gangopadhyay SS, Sen R (1999). Transcriptional activation by ETS and leucine zipper-containing basic helix-loop-helix proteins. Mol Cell Biol.

[11] Geisler S, Coller J (2013). RNA in unexpected places: long non-coding RNA functions in diverse cellular contexts. Nat Rev Mol Cell Biol.

[12] Tay Y, Rinn J, Pandolfi PP (2014). The multilayered complexity of ceRNA crosstalk and competition. Nature.

[13] Gu P, Chen X, Xie R, Xie W, Huang L, Dong W, Han J, Liu X, Shen J, Huang J (2019). A novel AR translational regulator lncRNA LBCS inhibits castration resistance of prostate cancer. Mol Cancer.

[14] Wang FW, Cao CH, Han K, Zhao YX, Cai MY, Xiang ZC, Zhang JX, Chen JW, Zhong LP, Huang Y (2019). APC-activated long noncoding RNA inhibits colorectal carcinoma pathogenesis through reduction of exosome production. J Clin Investig.

[15] Zhang Y, Pitchiaya S, Cieslik M, Niknafs YS, Tien JC, Hosono Y, Iyer MK, Yazdani S, Subramaniam S, Shukla SK (2018). Analysis of the androgen receptor-regulated lncRNA landscape identifies a role for ARLNC1 in prostate cancer progression. Nat Genet.

[16] Yin X, Wang B, Gan W, Zhuang W, Xiang Z, Han X, Li D (2019). TFE3 fusions escape from controlling of mTOR signaling pathway and accumulate in the nucleus promoting genes expression in Xp11.2 translocation renal cell carcinomas. J Exp Clin Cancer Res.

[17] Nititham J, Fergusson C, Palmer C, Liao W, Foerster J (2018). Candidate long-range regulatory sites acting on the IL17 pathway genes TRAF3IP2 and IL17RA are associated with psoriasis. Exp Dermatol.

[18] Alt EU, Barabadi Z, Pfnur A, Ochoa JE, Daneshimehr F, Lang LM, Lin D, Braun SE, Chandrasekar B, Izadpanah R (2018). TRAF3IP2, a novel therapeutic target in glioblastoma multiforme. Oncotarget.

[19] Zan XY, Li L (2019). Construction of lncRNA-mediated ceRNA network to reveal clinically relevant lncRNA biomarkers in glioblastomas. Oncol Lett.

[20] Anglard P, Trahan E, Liu S, Latif F, Merino MJ, Lerman MI, Zbar B, Linehan WM (1992). Molecular and cellular characterization of human renal cell carcinoma cell lines. Cancer Res.

[21] Zheng S, Yang L, Zou Y, Liang JY, Liu P, Gao G, Yang A, Tang H, Xie X (2020). Long non-coding RNA HUMT hypomethylation promotes lymphangiogenesis and metastasis via activating FOXK1 transcription in triple-negative breast cancer. J Hematol Oncol.

[22] Koirala P, Huang JG, Ho TT, Wu FT, Ding XF, Mo YY (2017). LncRNA AK023948 is a positive regulator of AKT. Nat Commun.

[23] Du H, Zhao Y, He J, Zhang Y, Xi H, Liu M, Ma J, Wu L (2016). YTHDF2 destabilizes m(6)A-containing RNA through direct recruitment of the CCR4-NOT deadenylase complex. Nat Commun.

[24] Li J, Chen Z, Chen F, Xie G, Ling Y, Peng Y, Lin Y, Luo N, Chiang CM, Wang H (2020). Targeted mRNA demethylation using an engineered dCas13b-ALKBH5 fusion protein. Nucleic Acids Res.

[25] Wilson C, Chen PJ, Miao Z, Liu DR (2020). Programmable m(6)A modification of cellular RNAs with a Cas13-directed methyltransferase. Nat Biotechnol.

[26] Li WD, Dong XS, He CJ, Tan G, Li ZY, Zhai B, Feng J, Jiang X, Liu C, Jiang HC (2019). LncRNA SNHG1 contributes to sorafenib resistance by activating the Akt pathway and is positively regulated by miR-21 in hepatocellular carcinoma cells. J Exp Clin Canc Res.

[27] Tang ZF, Kang BX, Li CW, Chen TX, Zhang ZM (2019). GEPIA2: an enhanced web server for large-scale expression profiling and interactive analysis. Nucleic Acids Res.

[28] Li JH, Liu S, Zhou H, Qu LH, Yang JH (2014). starBase v2.0: decoding miRNA-ceRNA, miRNA-ncRNA and protein-RNA interaction networks from large-scale CLIP-Seq data. Nucleic Acids Res.

[29] Hsieh MH, Chen YT, Chen YT, Lee YH, Lu J, Chien CL, Chen HF, Ho HN, Yu CJ, Wang ZQ (2017). PARP1 controls KLF4-mediated telomerase expression in stem cells and cancer cells. Nucleic Acids Res.

[30] Chen K, Wei Z, Zhang Q, Wu X, Rong R, Lu Z, Su J, de Magalhaes JP, Rigden DJ, Meng J (2019). WHISTLE: a high-accuracy map of the human N6-methyladenosine (m6A) epitranscriptome predicted using a machine learning approach. Nucleic Acids Res.

[31] Nishida N, Mimori K, Fabbri M, Yokobori T, Sudo T, Tanaka F, Shibata K, Ishii H, Doki Y, Mori M (2011). MicroRNA-125a-5p is an independent prognostic factor in gastric cancer and inhibits the proliferation of human gastric cancer cells in combination with trastuzumab. Clin Cancer Res.

[32] Avgeris M, Tsilimantou A, Levis PK, Tokas T, Sideris DC, Stravodimos K, Ardavanis A, Scorilas A (2018). Loss of GAS5 tumour suppressor lncRNA: an independent molecular cancer biomarker for short-term relapse and progression in bladder cancer patients. Br J Cancer.

[33] He W, Zhong G, Jiang N, Wang B, Fan X, Chen C, Chen X, Huang J, Lin T (2018). Long noncoding RNA BLACAT2 promotes bladder cancer-associated lymphangiogenesis and lymphatic metastasis. J Clin Investig.

[34] Ashouri A, Sayin VI, Van den Eynden J, Singh SX, Papagiannakopoulos T, Larsson E (2016). Pan-cancer transcriptomic analysis associates long non-coding RNAs with key mutational driver events. Nat Commun.

[35] Lanzos A, Carlevaro-Fita J, Mularoni L, Reverter F, Palumbo E, Guigo R, Johnson R (2017). Discovery of cancer driver long noncoding RNAs across 1112 tumour genomes: new candidates and distinguishing features. Sci Rep.

[36] Qian X, Zhao J, Yeung PY, Zhang QC, Kwok CK (2019). Revealing lncRNA structures and interactions by sequencing-based approaches. Trends Biochem Sci.

[37] Xu S, Wang P, Zhang J, Wu H, Sui S, Zhang J, Wang Q, Qiao K, Yang W, Xu H (2019). Ai-lncRNA EGOT enhancing autophagy sensitizes paclitaxel cytotoxicity via upregulation of ITPR1 expression by RNA-RNA and RNA-protein interactions in human cancer. Mol Cancer.

[38] Yang Y, Hsu PJ, Chen YS, Yang YG (2018). Dynamic transcriptomic m(6)A decoration: writers, erasers, readers and functions in RNA metabolism. Cell Res.

[39] Zhao Y, Shi Y, Shen H, Xie W (2020). m(6)A-binding proteins: the emerging crucial performers in epigenetics. J Hematol Oncol.

[40] Wang X, Lu Z, Gomez A, Hon GC, Yue Y, Han D, Fu Y, Parisien M, Dai Q, Jia G (2014). N6-methyladenosine-dependent regulation of messenger RNA stability. Nature.

[41] Dominissini D, Moshitch-Moshkovitz S, Schwartz S, Salmon-Divon M, Ungar L, Osenberg S, Cesarkas K, Jacob-Hirsch J, Amariglio N, Kupiec M (2012). Topology of the human and mouse m6A RNA methylomes revealed by m6A-seq. Nature.

[42] Park OH, Ha H, Lee Y, Boo SH, Kwon DH, Song HK, Kim YK (2019). Endoribonucleolytic cleavage of m(6)A-containing RNAs by RNase P/MRP complex. Mol Cell.

[43] Tan X, Banerjee P, Liu X, Yu J, Gibbons DL, Wu P, Scott KL, Diao L, Zheng X, Wang J (2018). The epithelial-to-mesenchymal transition activator ZEB1 initiates a prometastatic competing endogenous RNA network. J Clin Investig.

