# Correction to: Low expression of TRAF3IP2-AS1 promotes progression of *NONO-TFE3* translocation renal cell carcinoma by stimulating *N*^6^-methyladenosine of PARP1 mRNA and downregulating PTEN

**DOI:** 10.1186/s13045-021-01153-8

**Published:** 2021-09-14

**Authors:** Lei Yang, Yi Chen, Ning Liu, QianCheng Shi, Xiaodong Han, Weidong Gan, Dongmei Li

**Affiliations:** 1 Immunology and Reproduction Biology Laboratory & State Key Laboratory of Analytical Chemistry for Life Science, Medical School, Nanjing University, Nanjing, 210093 Jiangsu China; 2 Jiangsu Key Laboratory of Molecular Medicine, Nanjing University, Nanjing, 210093 Jiangsu China; 3 Department of Urology, Affiliated Drum Tower Hospital of Medical School, Nanjing University, Nanjing, 210008 Jiangsu China

## Correction to: J Hematol Oncol (2021) 14:46 10.1186/s13045-021-01059-5

The original article [1] contained errors in Fig. 8 whereby error bars and labels were mistakenly omitted. The figure has since been corrected.
