# Supplementary material for: Low expression of TRAF3IP2-AS1 promotes progression of NONO-TFE3 translocation renal cell carcinoma by stimulating N6-methyladenosine of PARP1 mRNA and downregulating PTEN
Source: J Hematol Oncol. 2021 Mar 19;14:46. doi: 10.1186/s13045-021-01059-5 (PMC7980631; doi:10.1186/s13045-021-01059-5)
Supplement: Supplementary file 1 — Additional file 1. Supplementary Materials and Primers. [file 13045_2021_1059_MOESM1_ESM.docx]

**Low expression of TRAF3IP2-AS1 promotes progression of *NONO-TFE3* translocation renal cell carcinoma by stimulating *N*^6^-methyladenosine of PARP1 mRNA and downregulating PTEN**

Lei Yang^1,2^, Yi Chen^1,2^, Ning Liu^1,2^, QianCheng Shi^1,2^, Xiaodong Han^1,2^, Weidong Gan^3*^, Dongmei Li^1,2*^

1. Immunology and Reproduction Biology Laboratory & State Key Laboratory of Analytical Chemistry for Life Science, Medical School, Nanjing University, Nanjing, Jiangsu 210093, China

2. Jiangsu Key Laboratory of Molecular Medicine, Nanjing University, Nanjing, Jiangsu 210093, China

3. Department of Urology, Affiliated Drum Tower Hospital of Medical School of

Nanjing University, Nanjing, Jiangsu 210008, China.

* Corresponding Author:

Dongmei Li, Immunology and Reproduction Biology Laboratory & State Key Laboratory of Analytical Chemistry for Life Science, Medical School, Nanjing University, Nanjing, Jiangsu 210093, China

Email: [lidm@nju.edu.cn](mailto:lidm@nju.edu.cn)

Weidong Gan, Department of Urology, Affiliated Drum Tower Hospital of Medical

School of Nanjing University, Nanjing, Jiangsu 210008, China.

Email: [gwd@nju.edu.cn](mailto:gwd@nju.edu.cn)

**Table S1.** Primers used for real-time PCR.

| Target  gene | Primer sequence (5’-3’) | | Size  (bp) |
| --- | --- | --- | --- |
|  | Forward | Reverse |  |
| TRAF3IP2-AS1 | TTTGGCGGCTATGCAGGATT | TGTCCATGTGGTATTGGGCA | 189 |
| PTEN | TTTGAAGACCATAACCCACCAC | ATTACACCAGTTCGTCCCTTTC | 134 |
| PARP1 | CGGAGTCTTCGGATAAGCTCT | TTTCCATCAAACATGGGCGAC | 136 |
| 18s rRNA | CAGCCACCCGAGATTGAGCA | TAGTAGCGACGGGCGGTGTG | 252 |
| GAPDH | AACGGATTTGGTCGTATTGGG | CCTGGAAGATGGTGATGGGAT | 211 |
| AKT1 | TCCTCCTCAAGAATGATGGCA | GTGCGTTCGATGACAGTGGT | 181 |
| CCND1 | CAATGACCCCGCACGATTTC | CATGGAGGGCGGATTGGAA | 146 |
| CCNB1 | AATAAGGCGAAGATCAACATGGC | TTTGTTACCAATGTCCCCAAGAG | 111 |
| BCL2 | GGTGGGGTCATGTGTGTGG | CGGTTCAGGTACTCAGTCATCC | 89 |
| MMP2 | GATACCCCTTTGACGGTAAGGA | CCTTCTCCCAAGGTCCATAGC | 112 |
| MMP9 | GGGACGCAGACATCGTCATC | TCGTCATCGTCGAAATGGGC | 139 |
| BIRC5 | AGGACCACCGCATCTCTACAT | AAGTCTGGCTCGTTCTCAGTG | 118 |
| METTL3 | TTGTCTCCAACCTTCCGTAGT | CCAGATCAGAGAGGTGGTGTAG | 145 |
| METTL14 | GAGTGTGTTTACGAAAATGGGGT | CCGTCTGTGCTACGCTTCA | 172 |
| WTAP | CTTCCCAAGAAGGTTCGATTGA | TCAGACTCTCTTAGGCCAGTTAC | 158 |
| FTO | GCTGCTTATTTCGGGACCTG | AGCCTGGATTACCAATGAGGA | 84 |
| ALKBH5 | CGGCGAAGGCTACACTTACG | CCACCAGCTTTTGGATCACCA | 128 |
| APBB2 | AGTCTTAGTGGAATGTCGTGTGC | ACGTTACCAGCATTAGGCTCG | 141 |
| UBE2G1 | AGGTGGTGTTTTTAAGGCTCATC | CATTTGGGTGCCAGATTTCTGTA | 95 |
| TET3 | TCCAGCAACTCCTAGAACTGAG | AGGCCGCTTGAATACTGACTG | 169 |
| NFIA | ATCCGACCCGAATATCGAGAG | AGTCAATTCTTCGCATCTTGCC | 109 |
| ARL4A | TGTTGTGGACTCTGTTGATGTC | AGCTCAGTTCACCCATTGCTA | 179 |
| ELAVL2 | CAACACCCTGAATGGATTGAGA | TTTTTGGAAGTCCGCTGACAT | 113 |
| CPEB4 | GGGCTGAATGGTGGAATAACG | GGGCGAGCATACTTCTGGA | 95 |
| ZNF609 | AAGGAGTGGGGACTTGTTCAG | CCCCTGCACTTCCATCATACT | 93 |
| SOX11 | AGGATTTGGATTCGTTCAGCG | AGGTCGGAGAAGTTCGCCT | 121 |
| TFE3 | TGCCTGTGTCAGGGAATCTG | CGACGCTCAATTAGGTTGTGAT | 184 |

**Table S2.** Primers used for ChIP assay.

| Target  promoter | Primer sequence (5’-3’) | | Size  (bp) |
| --- | --- | --- | --- |
|  | Forward | Reverse |  |
| TRAF3IP2-AS1 site1 | CAGAGCAGGCCCTACGAAC | CTCCGTGGATCGCTGCC | 286 |
| TRAF3IP2-AS1 site2 | GAAGCCCTCGAGCTTTCGTC | TTGCGTCCGTGACATCATCA | 123 |
| TRAF3IP2-AS1 site3 | ATGATGATGTCACGGACGCAA | ATGAGATCGGTGCTCCGCC | 295 |

**Table S3.** Primers used for m^6^A MeRIP-qRT-PCR analysis.

| Target  transcript | Primer sequence (5’-3’) | | Size  (bp) |
| --- | --- | --- | --- |
|  | Forward | Reverse |  |
| PARP1 primer1 | GAGAGGTAGCCGAGTCACAC | ACCCATCAGCAACTTAGCGG | 93 |
| PARP1 primer2 | AAAGGCTGGAGAGAGATTCTGTT | TTTTCCTTCCCTGGGGAAACC | 98 |

**Table S4.** Probes used for RNA FISH.

| Target transcript | Probe sequence (5’-3’) |
| --- | --- |
| TRAF3IP2-AS1 probe1 | CCTCTGCTGGATGTGAAATGGCGGT |
| TRAF3IP2-AS1 probe2 | CCCGCCTCCTCTGCTGGATGTGAAATGG |
| U6 probe | TTTGCGTGTCATCTTCG |
| 18s rRNA probe | CTGCCTTCCTTGGATGTGGGTAGCCGTTTC |

**Table S5.** SiRNA, shRNA and ASOs used for silencing target genes.

| Target  transcript | | Sequence (5’-3’) | |
| --- | --- | --- | --- |
|  |  | sense | antisense |
| TRAF3IP2-AS1 siRNA seq1 | | CAUUAGAAGUUAUAAUAAACA | UUUAUUAUAACUUCUAAUGUG |
| TRAF3IP2-AS1 siRNA seq2 | | GGAGUAUGUGCUUAAGGAAAU | UUCCUUAAGCACAUACUCCAA |
| TRAF3IP2-AS1 siRNA seq3 | | GAGCUGUGAUUCAAAUAUAAG | UAUAUUUGAAUCACAGCUCUG |
| METTL3 siRNA | | GCCAAGGAACAAUCCAUUGUU | AACAAUGGAUUGUUCCUUGGC |
| METTL14 siRNA | | CCAUGUACUUACAAGCCGAUA | UAUCGGCUUGUAAGUACAUGG |
| WTAP siRNA | | GUUAUGGCAAGAGAUGAGUUA | UAACUCAUCUCUUGCCAUAAC |
| FTO siRNA | | UCACCAAGGAGACUGCUAUUU | AAAUAGCAGUCUCCUUGGUGA |
| ALKBH5 siRNA | | GAAAGGCUGUUGGCAUCAAUA | UAUUGAUGCCAACAGCCUUUC |
| PARP1 siRNA | | CGACCUGAUCUGGAACAUCAA | UUGAUGUUCCAGAUCAGGUCG |
| PTEN siRNA | | CCACAAAUGAAGGGAUAUAAA | UUUAUAUCCCUUCAUUUGUGG |
| TFE3 shRNA | CAGCTCCGAATTCAGGAACTACTCGAGTAGTTCCTGAATTCGGAGCTG | | |
| TRAF3IP2-AS1 ASO | G^M^G^M^A^M^G^M^T^D^A^D^T^D^G^D^T^D^G^D^C^D^T^D^T^D^A^D^A^D^G^D^G^M^A^M^A^M^A^M^ | | |

**Table S6.** Guide RNA used for SAM system and targeted RNA methylation system.

| Target promoter | gRNA sequence (5’-3’) |
| --- | --- |
| TRAF3IP2-AS1 gRNA1 | AGGGGGAGAAAGCCCCTTCT |
| TRAF3IP2-AS1 gRNA2 | GGGATCGATGACTCGGCTGG |
| TRAF3IP2-AS1 gRNA3 | GGGGTGTGTGTGGCGAAGGG |
| Target transcript | gRNA sequence (5’-3’) |
| PARP1 gRNA1 | CAACAGAATCTCTCTCCAGCCTT |
| PARP1 gRNA2 | GCAGACATTCTAACGAAGCTTGG |
| PARP1 gRNA3 | ATGCAACAGAATCTCTCTCCAGC |

**Table S7.** Primary antibodies used in this study.

| Source | Primary antibodies | Catalog no. | Working dilution |
| --- | --- | --- | --- |
| Sigma-Aldrich | Anti-TFE3 antibody produced in rabbit | HPA023881 | IHC: 1:200 ChIP: 5μg |
| ProteinTech | Anti-TFE3 antibody produced in rabbit | 14480-1-AP | WB: 1:2000 |
| Santa Cruz Biotechnology | Anti-PARP1 antibody produced in mouse | sc-8007 | WB: 1:1000 |
| Santa Cruz Biotechnology | Anti-PTEN antibody produced in mouse | sc-7974 | WB: 1:1000 |
| ProteinTech | Anti-METTL3 antibody produced in rabbit | 15073-1-AP | WB: 1:1000 RIP: 5μg |
| ProteinTech | Anti-METTL14 antibody produced in rabbit | 26158-1-AP | WB: 1:1500 |
| ProteinTech | Anti-WTAP antibody produced in mouse | 60188-1-Ig | WB: 1:5000 |
| ProteinTech | Anti-FTO antibody produced in rabbit | 27226-1-AP | WB: 1:1000 |
| ProteinTech | Anti-ALKBH5 antibody produced in rabbit | 16837-1-AP | WB: 1:2000 |
| ProteinTech | Anti-YTHDF1 antibody produced in rabbit | 17479-1-AP | WB: 1:1000 |
| ProteinTech | Anti-YTHDF2 antibody produced in rabbit | 24744-1-AP | WB: 1:2500 RIP: 5μg |
| ProteinTech | Anti-Flag antibody produced in rabbit | 80010-1-RR | WB: 1:2500 ChIP: 5μg |
| ProteinTech | Anti-GFP antibody produced in rabbit | 50430-2-AP | WB: 1:2500 RIP: 5μg |
| ProteinTech | Anti-ACTB antibody produced in mouse | 60008-1-Ig | WB: 1:8000 |
| Abcam | Anti-m^6^A antibody produced in mouse | ab151230 | RIP: 5μg |
| Cell Signaling Technology | Anti-AGO2 antibody produced in rabbit | 2897 | RIP: 5μg |
