# Supplementary material for: Low expression of TRAF3IP2-AS1 promotes progression of NONO-TFE3 translocation renal cell carcinoma by stimulating N6-methyladenosine of PARP1 mRNA and downregulating PTEN
Source: J Hematol Oncol. 2021 Mar 19;14:46. doi: 10.1186/s13045-021-01059-5 (PMC7980631; doi:10.1186/s13045-021-01059-5)
Supplement: Supplementary file 2 — Additional file 2. Supplementary Figures. [file 13045_2021_1059_MOESM2_ESM.docx]

**Low expression of TRAF3IP2-AS1 promotes progression of *NONO-TFE3* translocation renal cell carcinoma by stimulating *N*^6^-methyladenosine of PARP1 mRNA and downregulating PTEN**

Lei Yang^1,2^, Yi Chen^1,2^, Ning Liu^1,2^, QianCheng Shi^1,2^, Xiaodong Han^1,2^, Weidong Gan^3*^, Dongmei Li^1,2*^

1. Immunology and Reproduction Biology Laboratory & State Key Laboratory of Analytical Chemistry for Life Science, Medical School, Nanjing University, Nanjing, Jiangsu 210093, China

2. Jiangsu Key Laboratory of Molecular Medicine, Nanjing University, Nanjing, Jiangsu 210093, China

3. Department of Urology, Affiliated Drum Tower Hospital of Medical School of

Nanjing University, Nanjing, Jiangsu 210008, China.

* Corresponding Author:

Dongmei Li, Immunology and Reproduction Biology Laboratory & State Key Laboratory of Analytical Chemistry for Life Science, Medical School, Nanjing University, Nanjing, Jiangsu 210093, China

Email: [lidm@nju.edu.cn](mailto:lidm@nju.edu.cn)

Weidong Gan, Department of Urology, Affiliated Drum Tower Hospital of Medical

School of Nanjing University, Nanjing, Jiangsu 210008, China.

Email: [gwd@nju.edu.cn](mailto:gwd@nju.edu.cn)


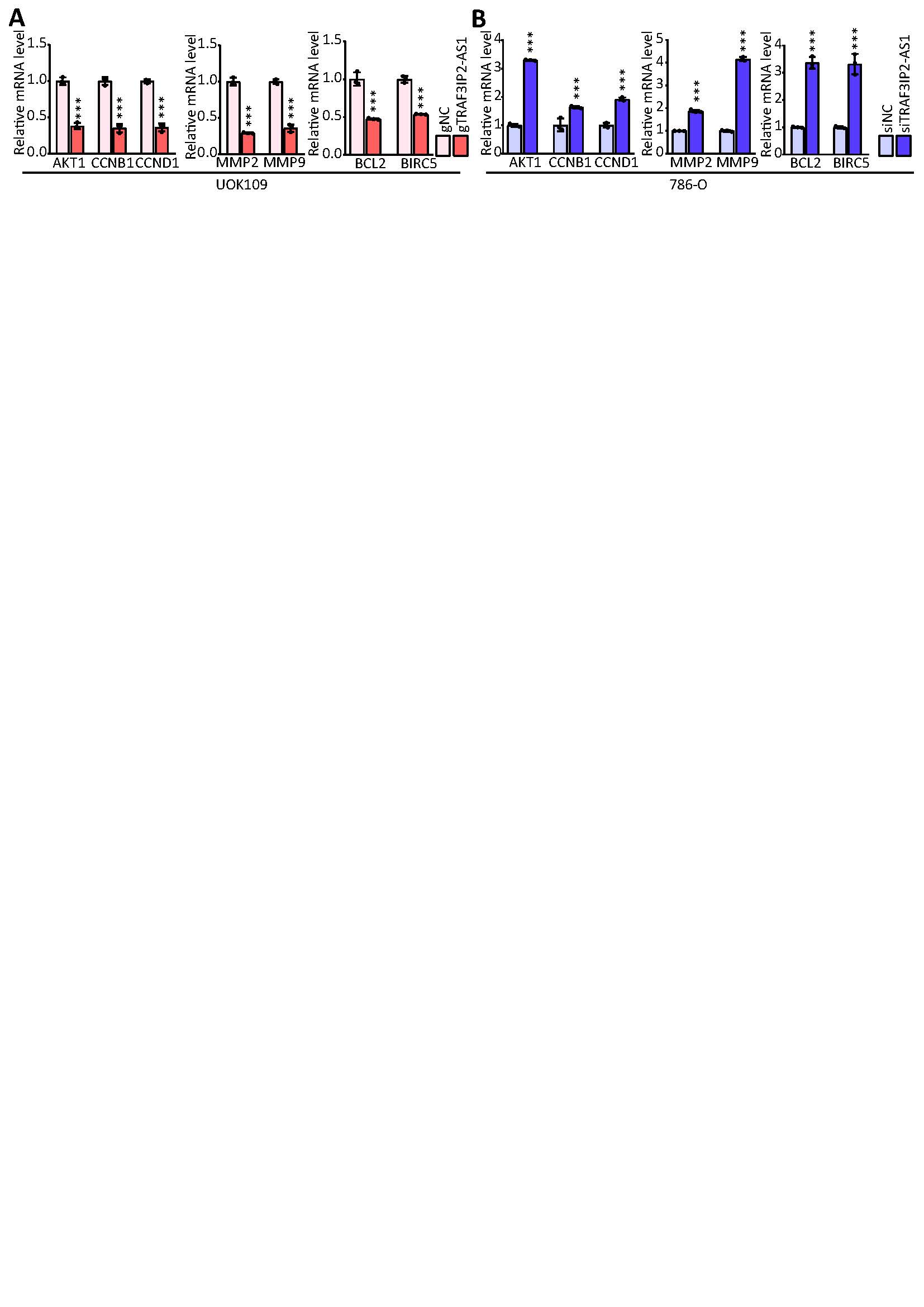


**Figure S1.** TRAF3IP2-AS1mediates the expression of genes related with cell proliferation, cell cycle, apoptosis, migration and invasion in UOK109 **(A)** and 786-O **(B)**. The data are presented as the mean ± SD, ****P*< 0.001


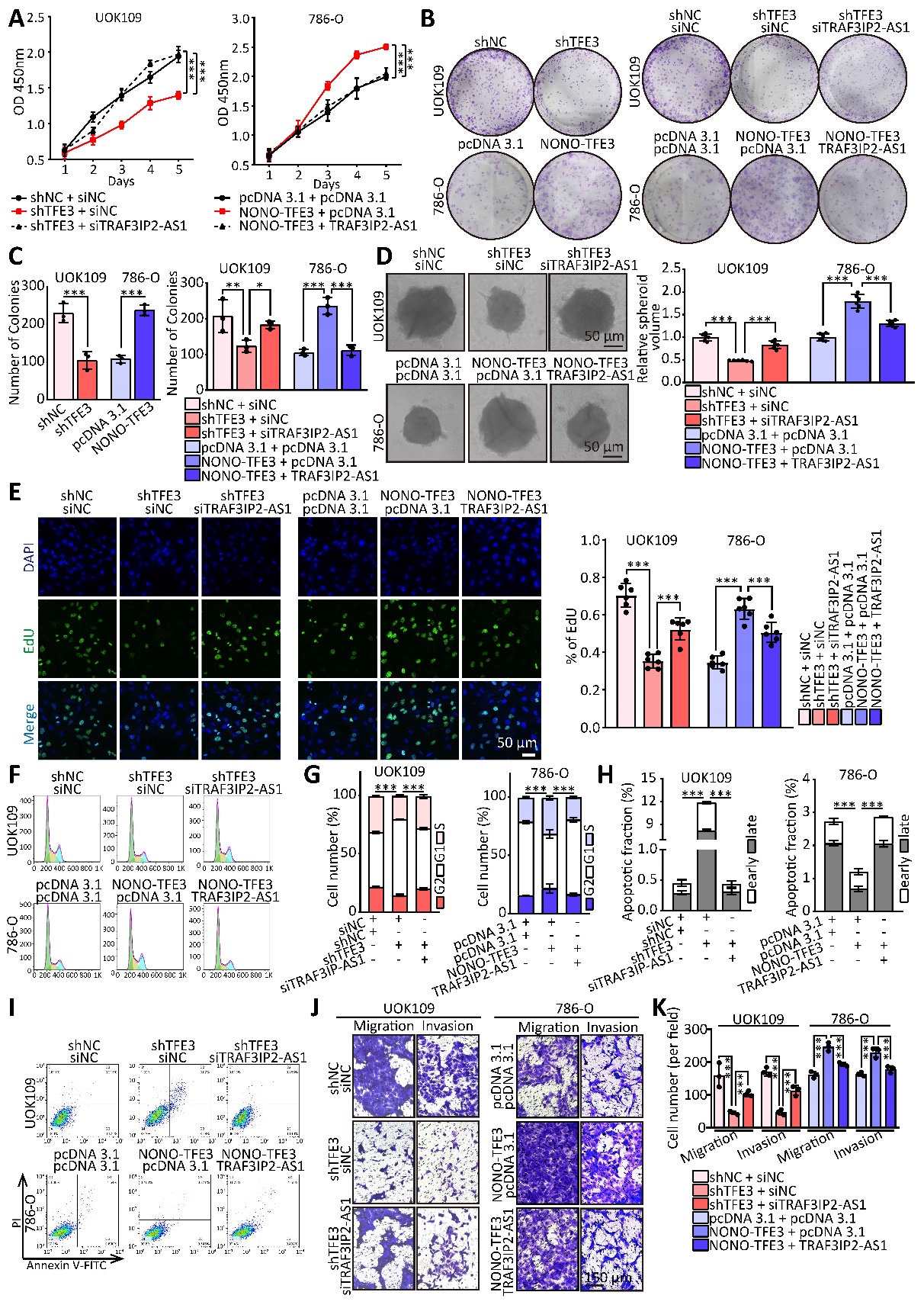


**Figure S2.** NONO-TFE3 promotes tumor progression through TRAF3IP2-AS1. **(A)** Cell viability of UOK109 and 786-O cells was determined using CCK-8 assays after transfection for 48h. **(B-D)** A colony formation and tumor sphere formation assay were used to determine the colony and tumor sphere formation ability of UOK109 and 786-O cells co-transfected with indicated vectors or siRNAs/ASO. **(E)** The cell viability of UOK109 and 786-O cells were detected after co-transfection with indicated vectors or siRNAs by EdU. **(F-G)** Cell cycle analysis was performed using flow cytometry in cells transfected with indicated vectors or siRNAs. **(H-I)** Apoptosis rate was tested using flow cytometry after transfection of UOK109 and 786-O cells for 48 h. **(J-K)** The invasion and migration abilities of UOK109 and 786-O cells co-transfected with indicated vectors or siRNAs were monitored by Transwell migration and invasion assays. The data are presented as the mean ± SD, **P*< 0.05, ***P*< 0.01, ****P*< 0.001


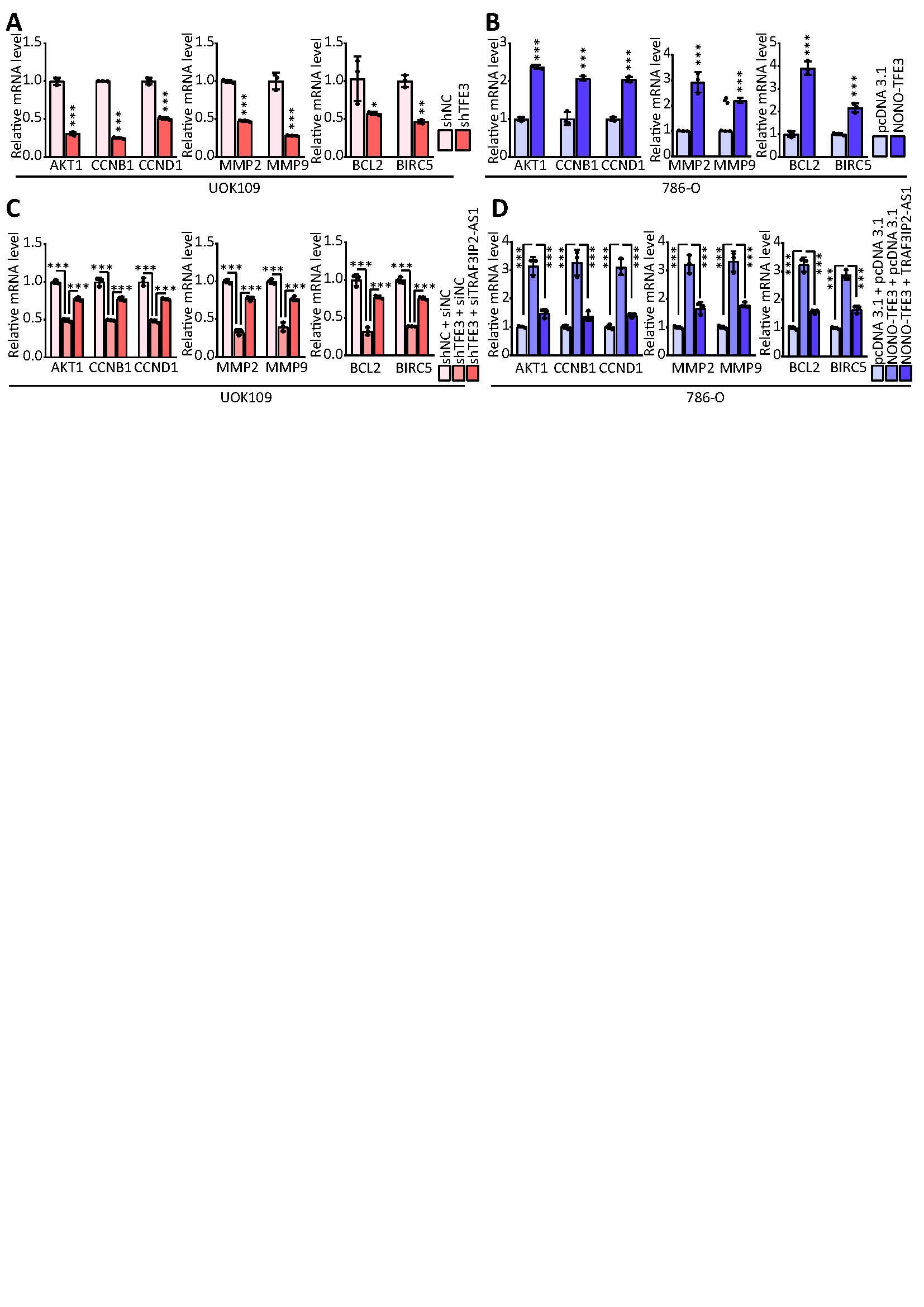


**Figure S3.** TFE3 mediates the expression of genes related with cell proliferation, cell cycle, apoptosis, migration and invasion through TRAF3IP2-AS1 in UOK109 **(A, C)** and 786-O **(B, D)**. The data are presented as the mean ± SD, **P*< 0.05, ****P*< 0.001


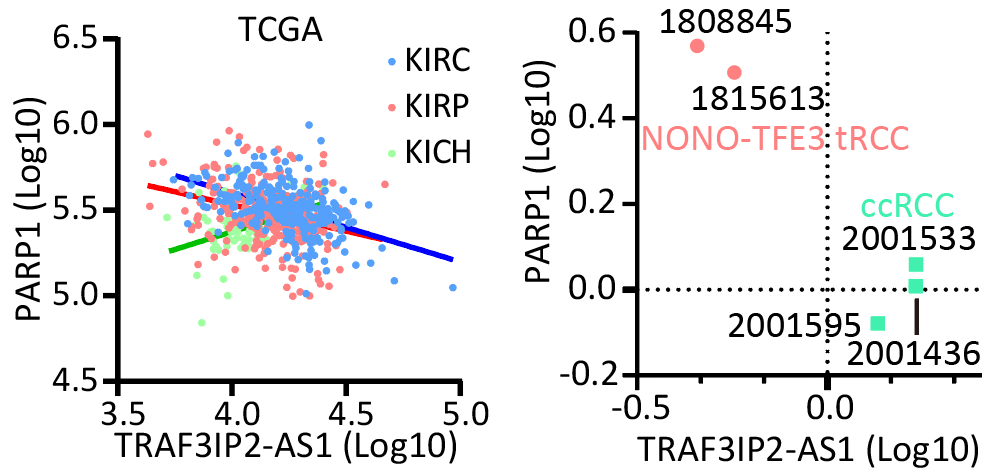


**Figure S4.** The correlation between TRAF3IP2-AS1 and PARP1.


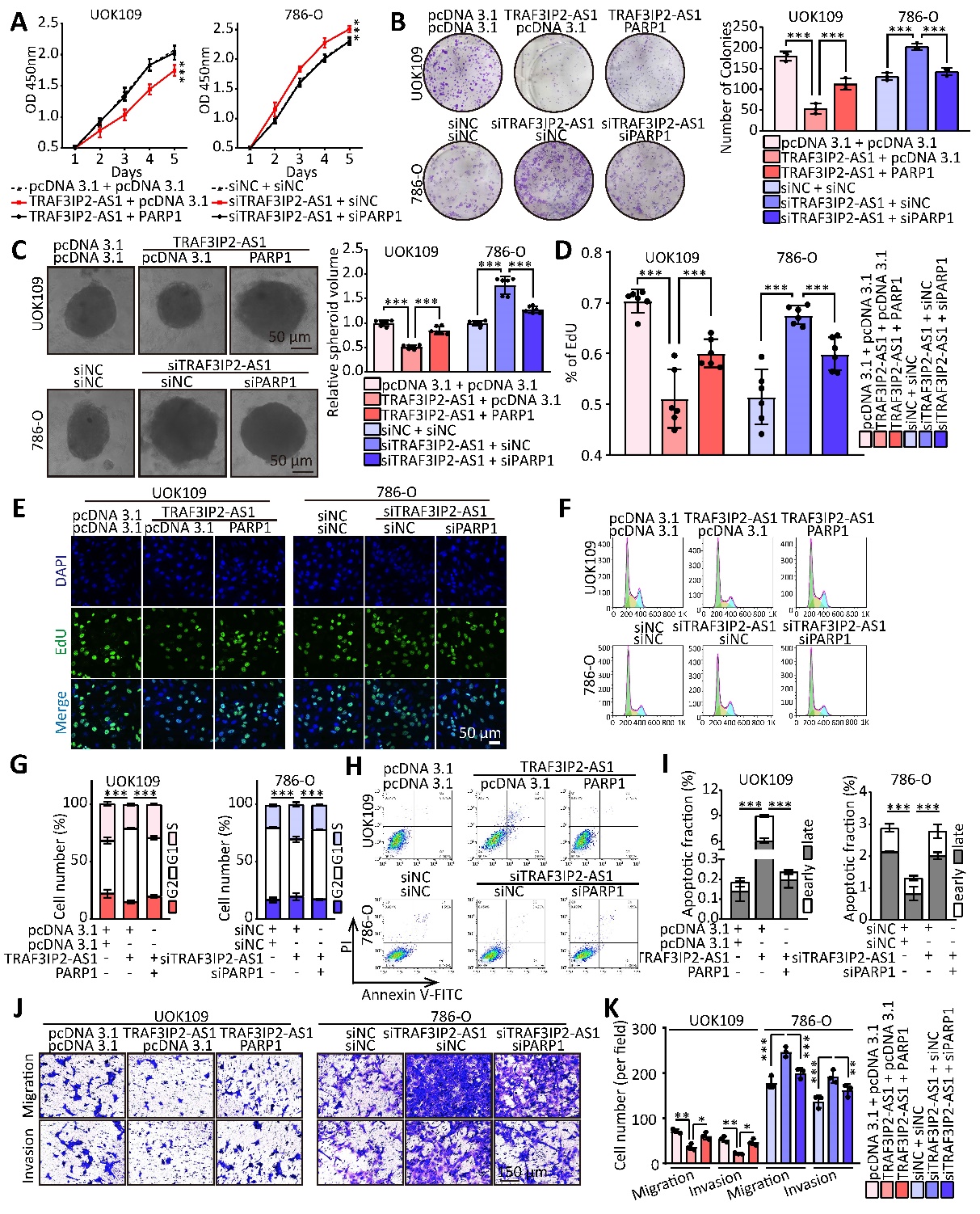


**Figure S5.** TRAF3IP2-AS1 mediates *NONO-TFE3* tRCC progression through PARP1. **(A)** Cell viability of UOK109 and 786-O cells was determined using CCK-8 assays after transfection for 48h. **(B-C)** A colony formation and tumor sphere formation assay were used to determine the colony and tumor sphere formation ability of UOK109 and 786-O cells co-transfected with indicated vectors or siRNAs/ASO. **(D-E)** The cell viability of UOK109 and 786-O cells were detected after co-transfection with indicated vectors or siRNAs/ASO by EdU. **(F-G)** Cell cycle analysis was performed using flow cytometry in cells transfected with indicated vectors or siRNAs/ASO. **(H-I)** Apoptosis rate was tested using flow cytometry after transfection of UOK109 and 786-O cells for 48 h. **(J-K)** The invasion and migration abilities of UOK109 and 786-O cells co-transfected with indicated vectors or siRNAs/ASO were monitored by Transwell migration and invasion assays. The data are presented as the mean ± SD, **P*< 0.05, ***P*< 0.01, ****P*< 0.001


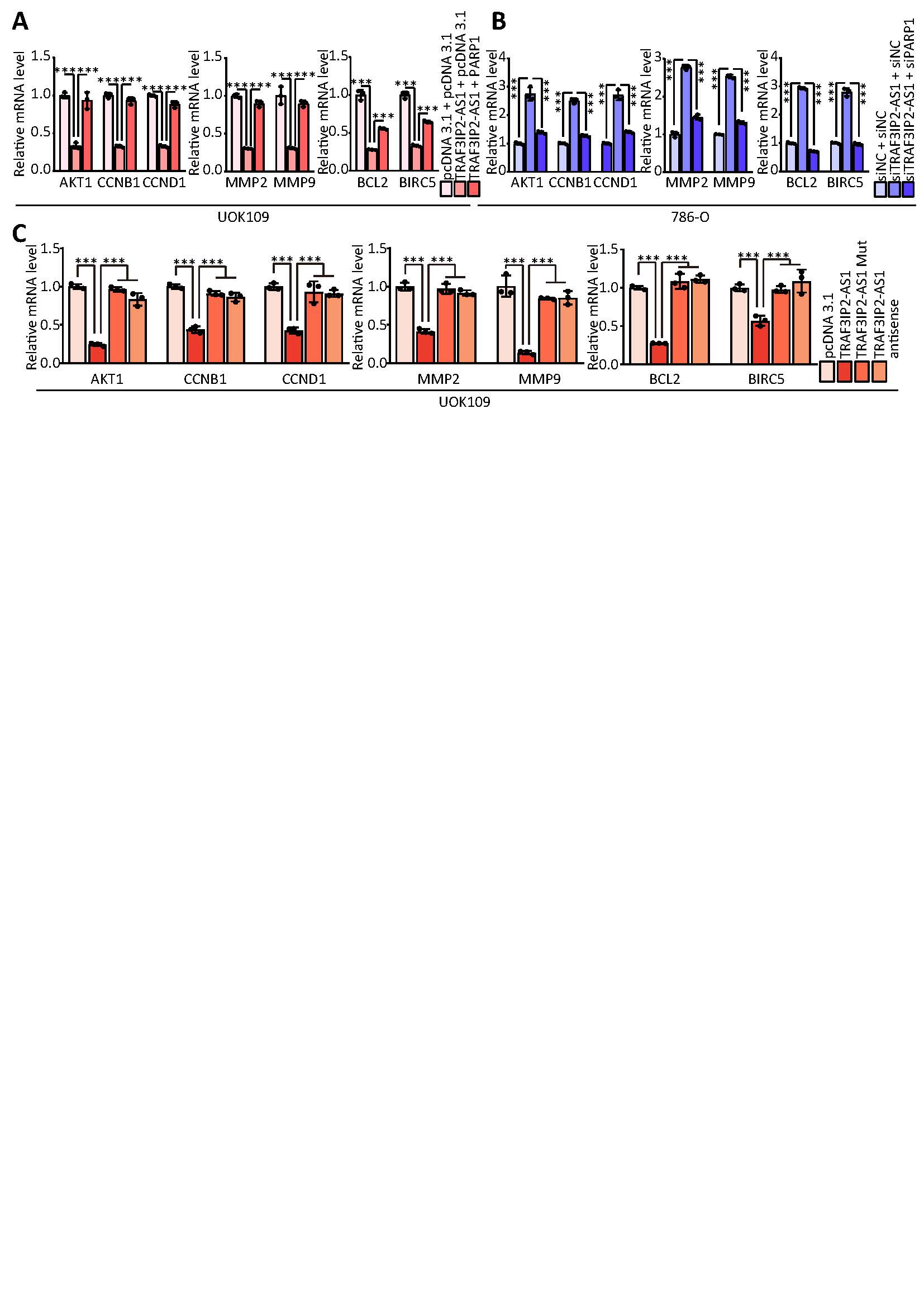


**Figure S6.** TRAF3IP2-AS1 mediates the expression of genes related with cell proliferation, cell cycle, apoptosis, migration and invasion through PARP1 in UOK109 **(A, C)** and 786-O **(B)**. The data are presented as the mean ± SD, ****P*< 0.001


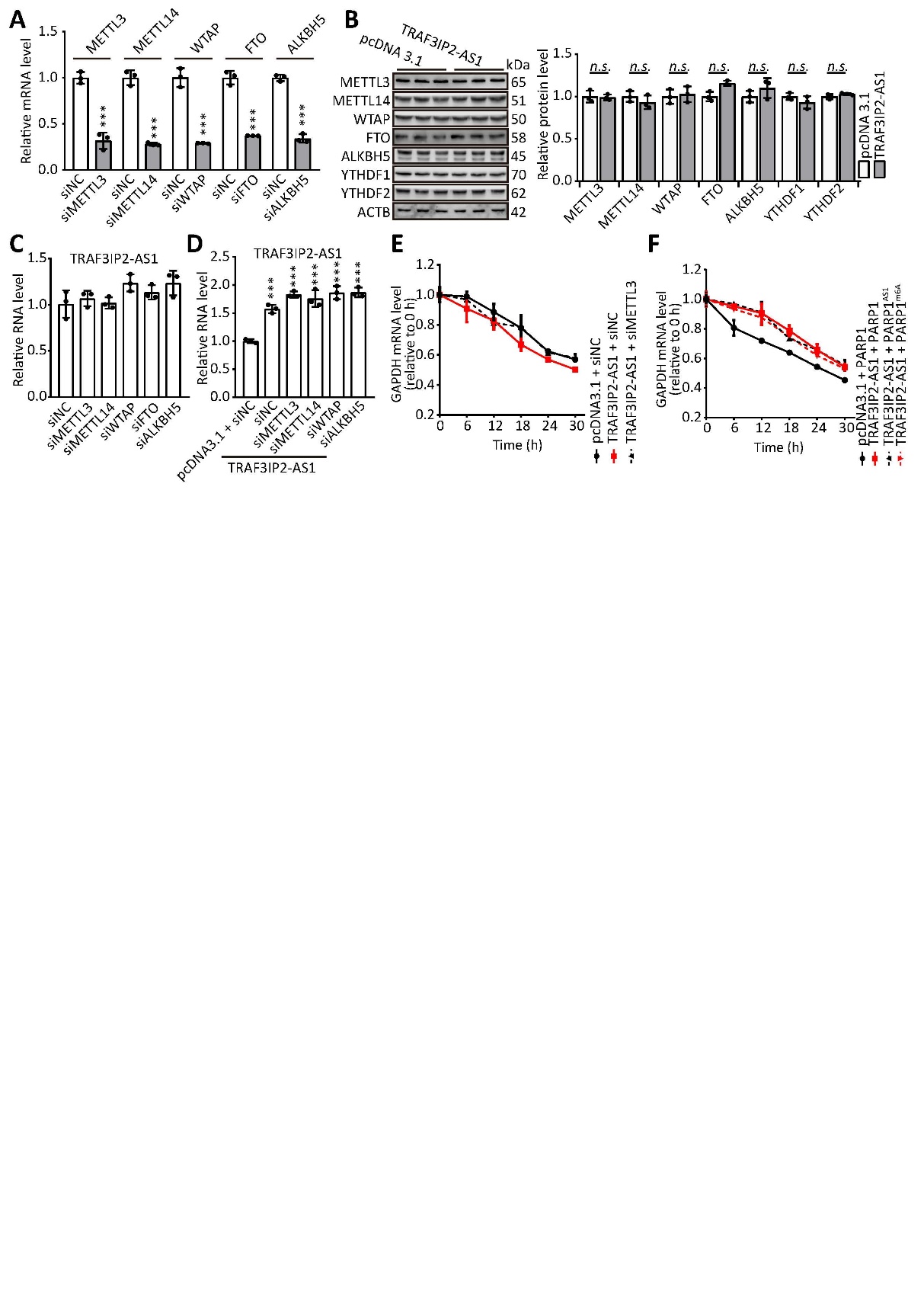


**Figure S7.** TRAF3IP2-AS1 accelerates the decay of PARP1 mRNA by recruitment of m^6^A methyltransferase complex. **(A)** The mRNA level of METTLL3, METTL14, WTAP, FTO and ALKBH5 were detected by qRT-PCR after transfected with indicated siRNAs. **(B)** The protein level of METTLL3, METTL14, WTAP, FTO, ALKBH5 and YTHDF1/2 were detected by Western blot after transfected with TRAF3IP2-AS1 plasmid. **(C-D)** The RNA level of TRAF3IP2-AS1 was detected by qRT-PCR after transfected with indicated siRNAs/ASO. **(E-F)** The stability of GAPDH mRNA in cells transfected with indicated plasmid and siRNAs after treatment with α-amanitin. The data are presented as the mean ± SD, ****P*< 0.001


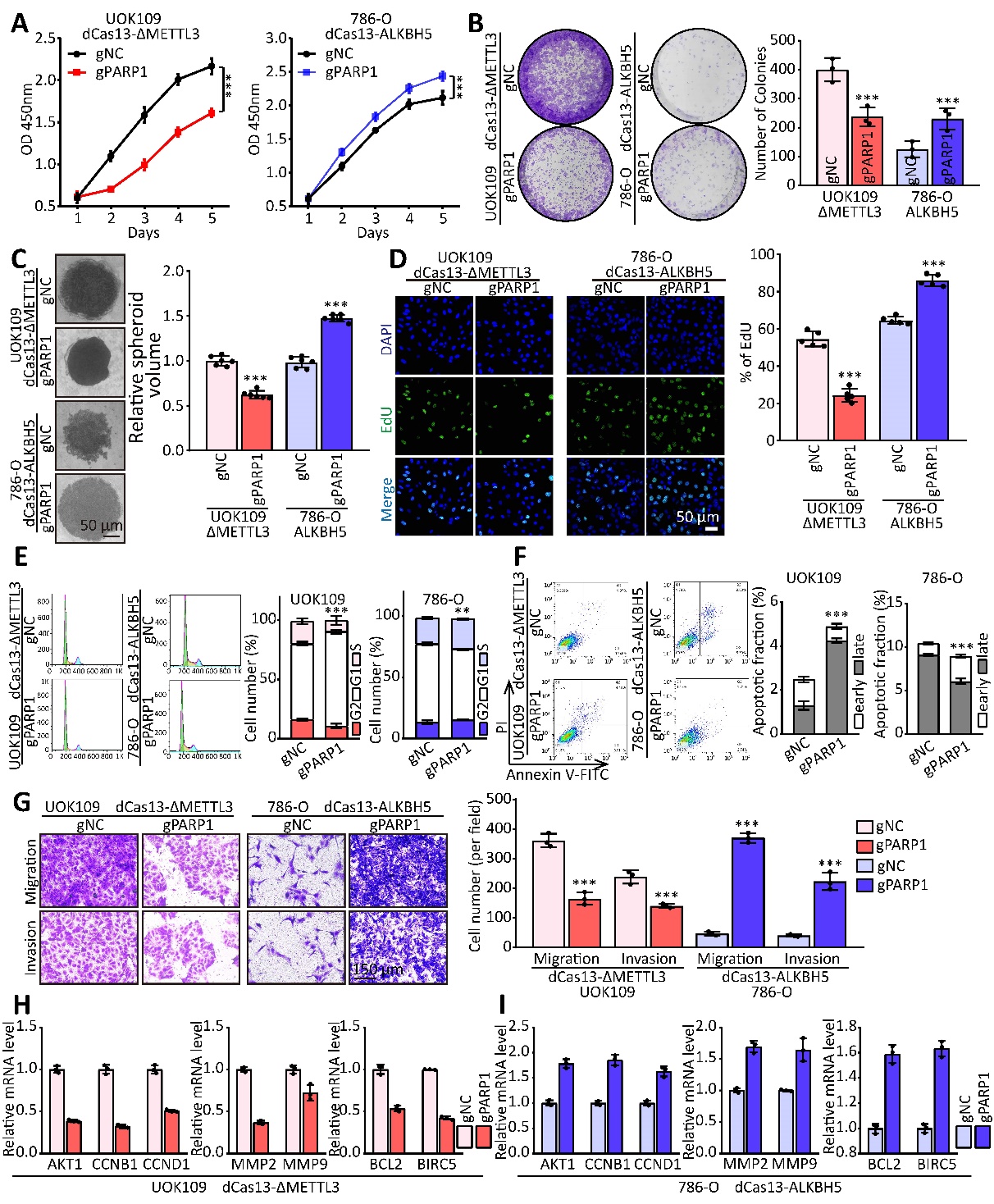


**Figure S8.** The level of m^6^A modification of PARP1 mRNA mediates *NONO-TFE3* tRCC progression. **(A)** Cell viability of UOK109 and 786-O cells was determined using CCK-8 assays after transfection for 48h. **(B-C)** A colony formation and tumor sphere formation assay were used to determine the colony and tumor sphere formation ability of UOK109 and 786-O cells co-transfected with indicated dCas13 fusion and gRNA. **(D)** The cell viability of UOK109 and 786-O cells were detected after co-transfection with indicated dCas13 fusion and gRNA by EdU. **(E)** Cell cycle analysis was performed using flow cytometry in cells transfected with indicated indicated dCas13 fusion and gRNA. **(F)** Apoptosis rate was tested using flow cytometry after transfection of UOK109 and 786-O cells for 48 h. **(G)** The invasion and migration abilities of UOK109 and 786-O cells co-transfected with indicated dCas13 fusion and gRNA were monitored by Transwell migration and invasion assays. **(H-I)** The level of m^6^A modification of PARP1 mRNA mediates the expression of genes related with cell proliferation, cell cycle, apoptosis, migration and invasion through PARP1 in UOK109 **(H)** and 786-O **(I)**.The data are presented as the mean ± SD, ***P*< 0.01, ****P*< 0.001


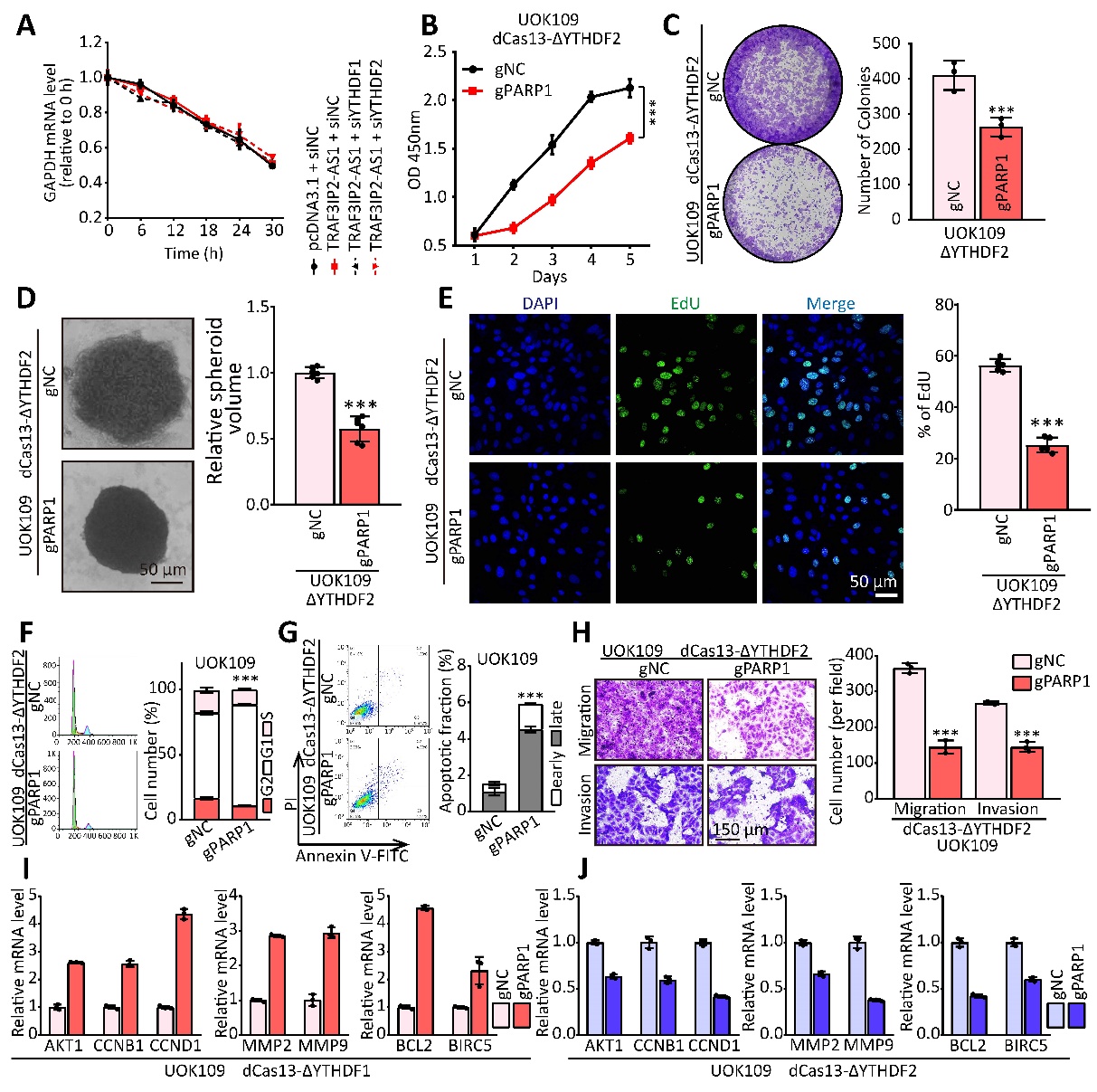


**Figure S9.** YTHDF2 mediates the decay of PARP1 mRNA to regulate NONO-TFE3 tRCC progression. **(A)** The stability of GAPDH mRNA in cells transfected with indicated plasmid and siRNAs after treatment with α-amanitin. **(B)** Cell viability of UOK109 and 786-O cells was determined using CCK-8 assays after transfection for 48h. **(C-D)** A colony formation and tumor sphere formation assay were used to determine the colony and tumor sphere formation ability of UOK109 and 786-O cells co-transfected with indicated dCas13 fusion and gRNA. **(E)** The cell viability of UOK109 and 786-O cells were detected after co-transfection with indicated dCas13 fusion and gRNA by EdU. **(F)** Cell cycle analysis was performed using flow cytometry in cells transfected with indicated indicated dCas13 fusion and gRNA. **(G)** Apoptosis rate was tested using flow cytometry after transfection of UOK109 and 786-O cells for 48 h. **(H)** The invasion and migration abilities of UOK109 and 786-O cells co-transfected with indicated dCas13 fusion and gRNA were monitored by Transwell migration and invasion assays. **(I-J)** The level of m^6^A modification of PARP1 mRNA mediates the expression of genes related with cell proliferation, cell cycle, apoptosis, migration and invasion through PARP1 in UOK109 **(I)** and 786-O **(J)**.The data are presented as the mean ± SD, ****P*< 0.001


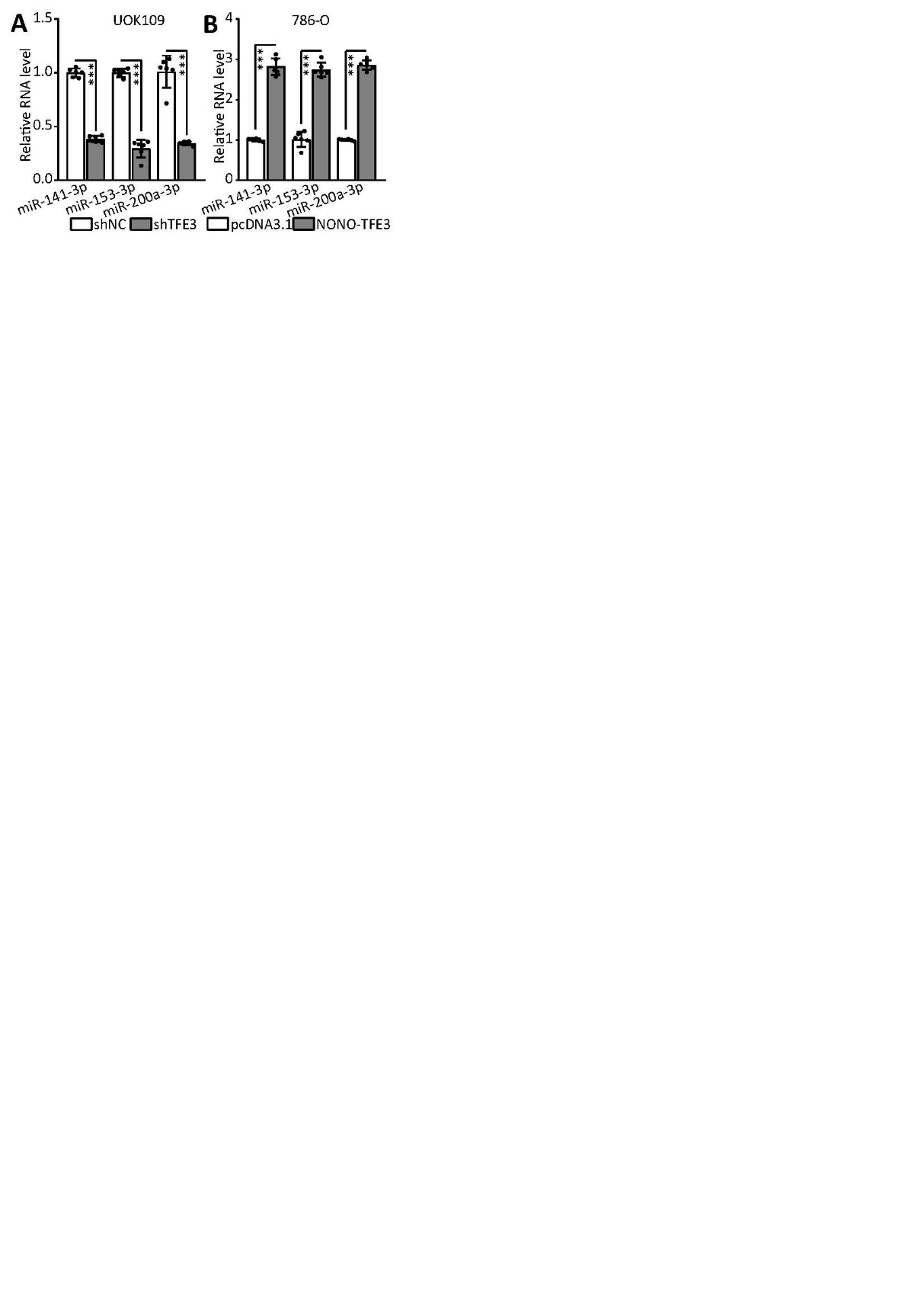


**Figure S10.** The RNA level of miRNAs were detected by qRT-PCR after transfected with indicated shRNA and plasmid. The data are presented as the mean ± SD, ****P*< 0.001


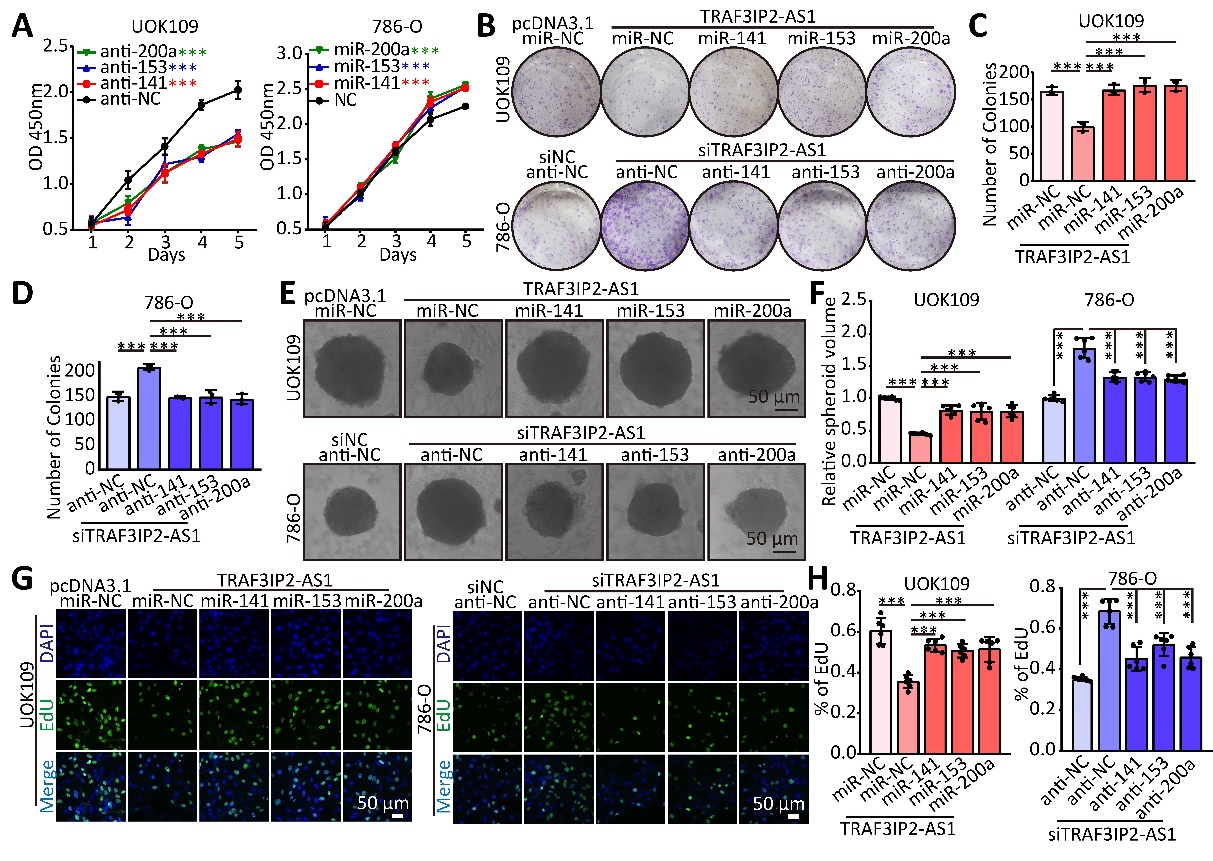


**Figure S11.** TRAF3IP2-AS1 acts as miRNA sponge to mediate cell proliferation. **(A)** Cell viability of UOK109 and 786-O cells was determined using CCK-8 assays after transfection for 48h. **(B-F)** A colony formation and tumor sphere formation assay were used to determine the colony and tumor sphere formation ability of UOK109 and 786-O cells co-transfected with indicated vectors, siRNA or miRNAs inhibitor. **(G-H)** The cell proliferation of UOK109 and 786-O cells were detected after co-transfection with indicated vectors, siRNA or miRNAs inhibitor by EdU. The data are presented as the mean ± SD, **P*< 0.05, ****P*< 0.001


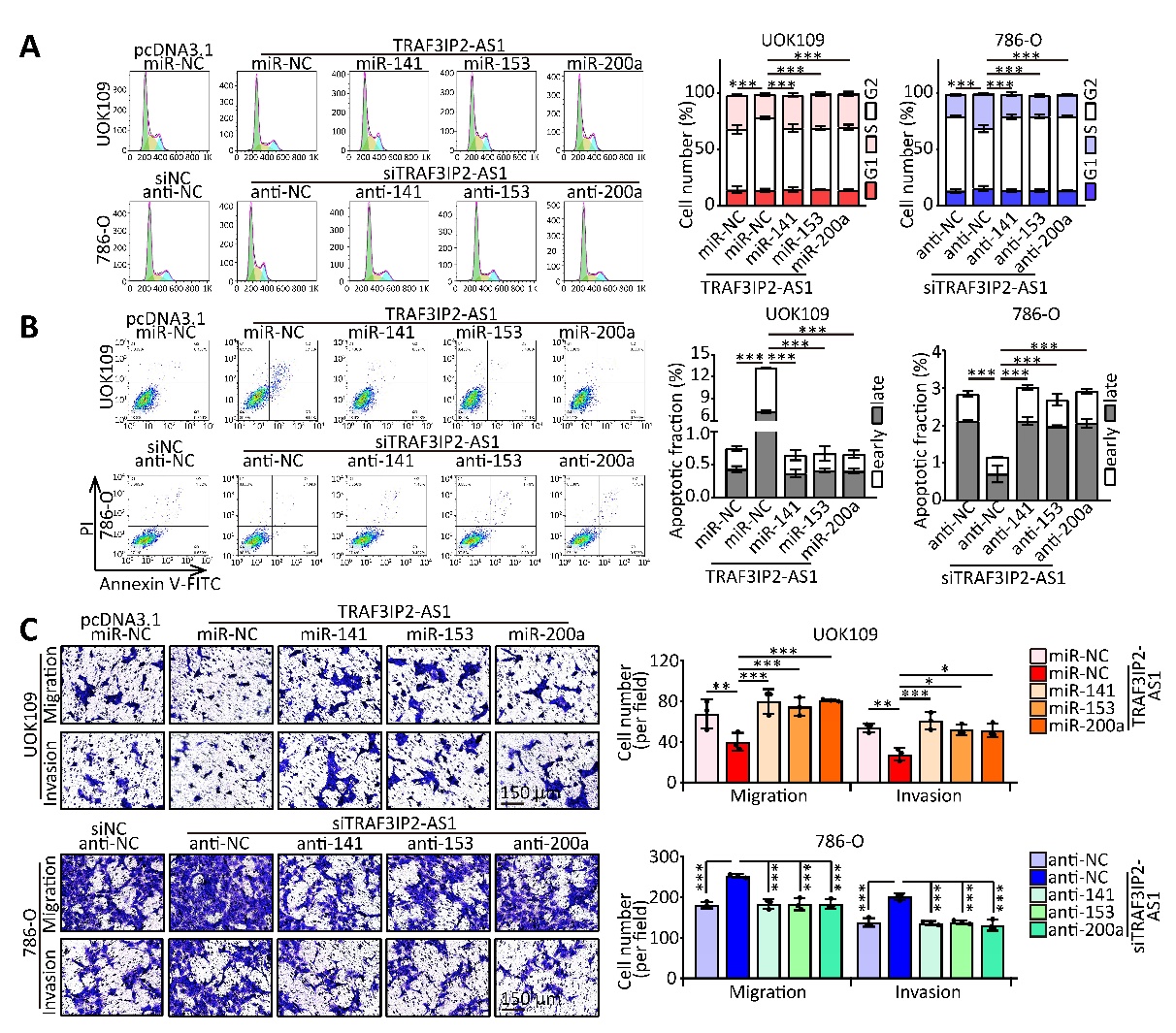


**Figure S12.** TRAF3IP2-AS1 acts as miRNA sponge to mediate cell cycle, apoptosis, migration and invasion. **(A)** Cell cycle analysis was performed using flow cytometry in cells transfected with indicated vectors, siRNA or miRNAs inhibitor. **(B)** Apoptosis rate was tested using flow cytometry after transfection of UOK109 and 786-O cells for 48 h. **(C)** The invasion and migration abilities of UOK109 and 786-O cells co-transfected with indicated vectors, siRNA or miRNAs inhibitor were monitored by Transwell migration and invasion assays. The data are presented as the mean ± SD, **P*< 0.05, ***P*< 0.01, ****P*< 0.001


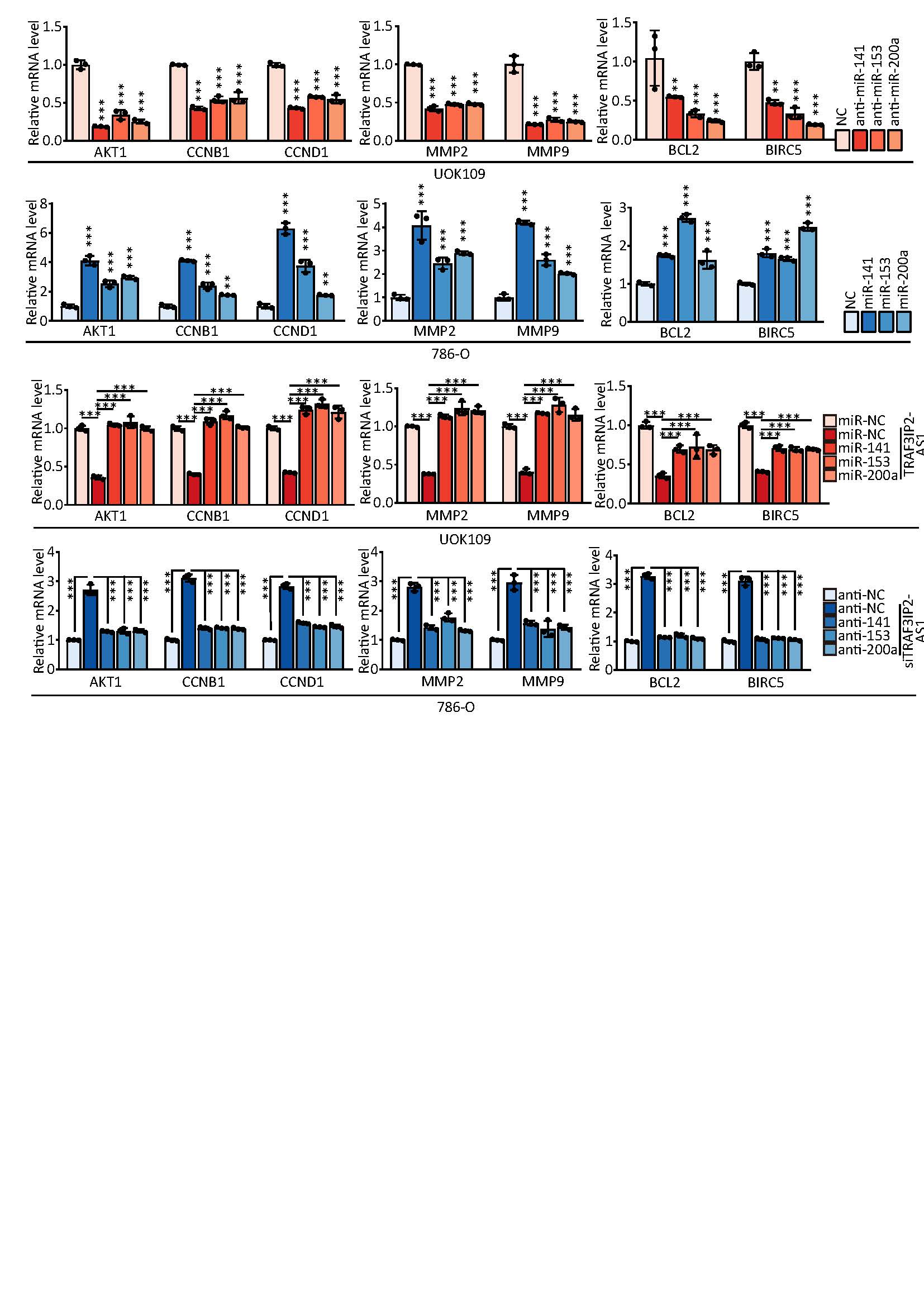


**Figure S13.** TRAF3IP2-AS1 mediates the expression of genes related with cell proliferation, cell cycle, apoptosis, migration and invasion through miR-141, -153 and -200a in UOK109 and 786-O. The data are presented as the mean ± SD, ***P*< 0.01, ****P*< 0.001


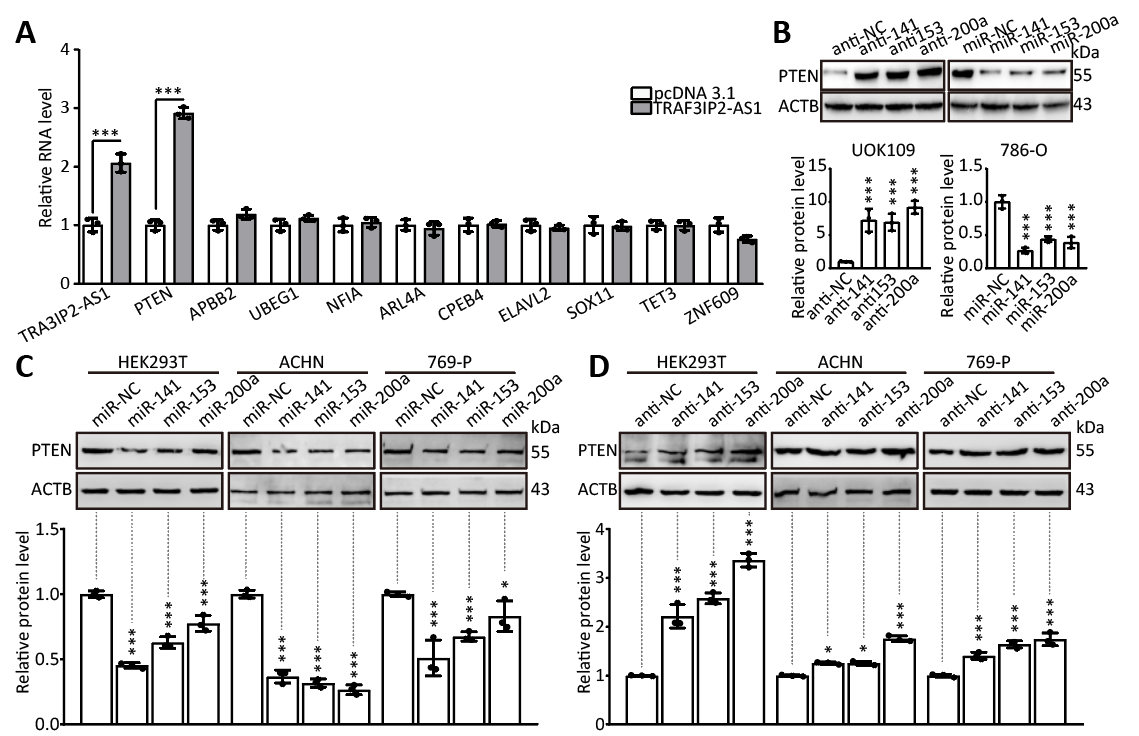


**Figure S14.** TRAF3IP2-AS1 modulates the expression of PTEN through post-transcriptional regulation of miRNAs. **(A)** The mRNA level of potential target genes of miRNAs were detected by qRT-PCR after overexpressed of TRAF3IP2-AS1. **(B-D)** The protein level of PTEN was detected by Western blot after transfected with indicated miRNAs inhibitor or plasmid. The data are presented as the mean ± SD, **P*< 0.05, ***P*< 0.01, ****P*< 0.001


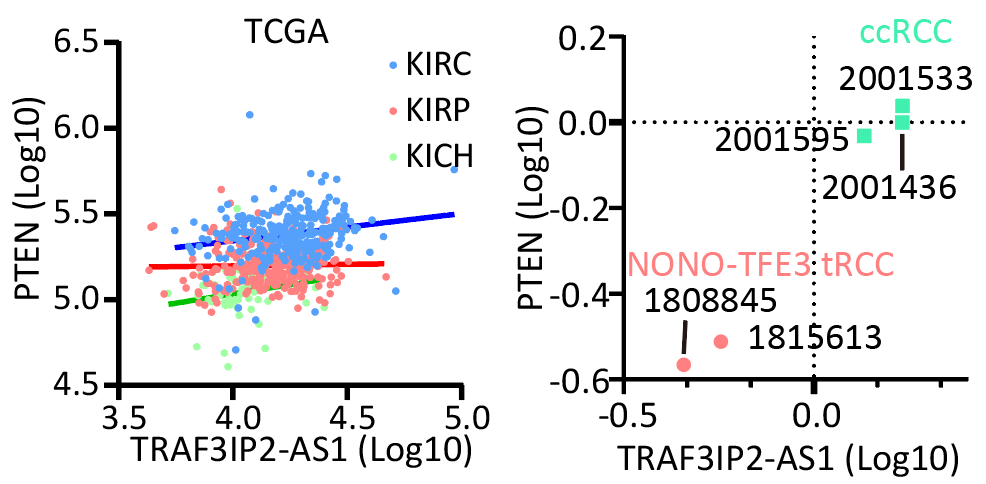


**Figure S15.** The correlation between TRAF3IP2-AS1 and PTEN.


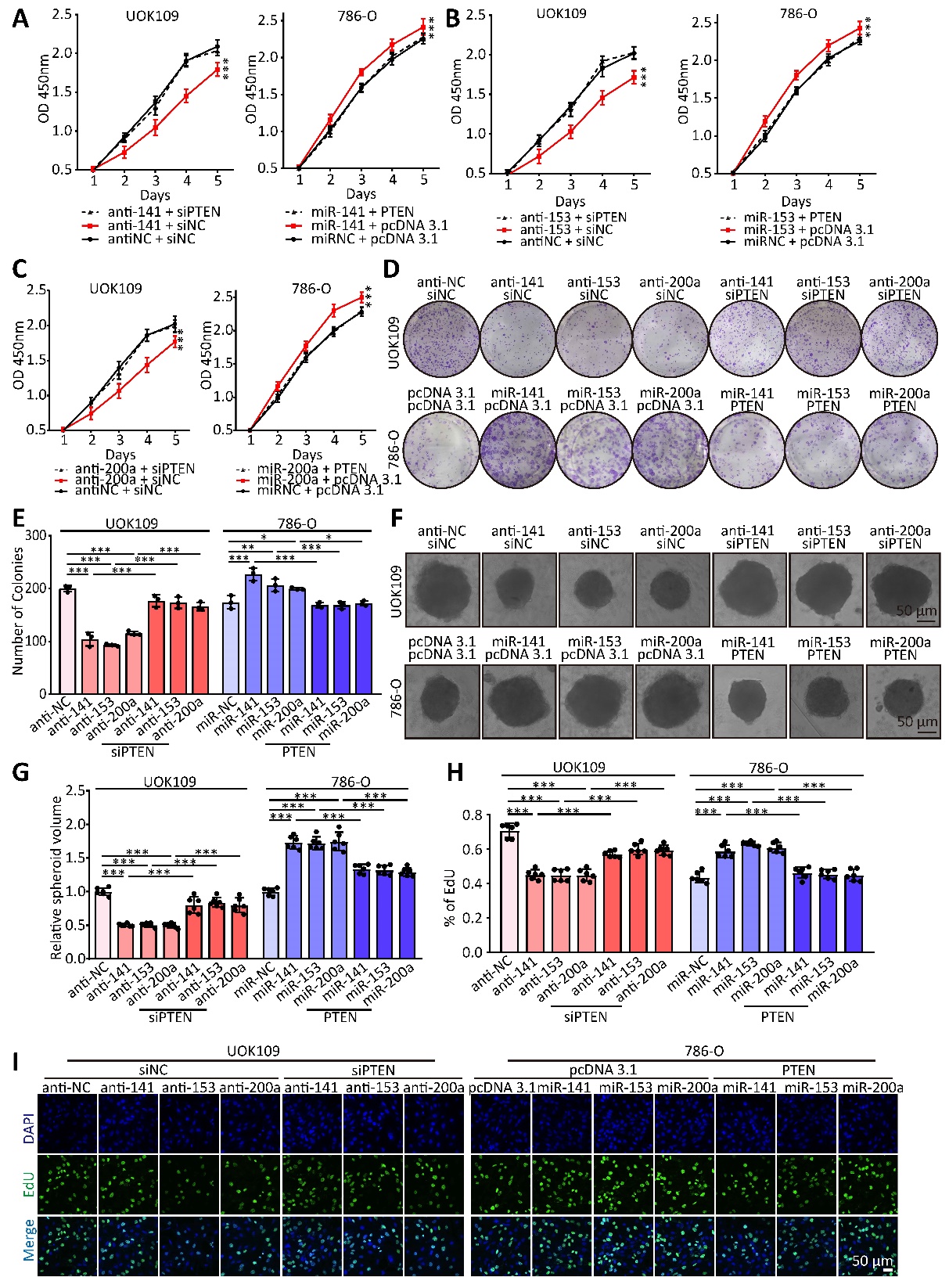


**Figure S16.** MiRNAs promote the cell proliferation of *NONO-TFE3* tRCC by targeting PTEN. **(A-C)** Cell viability of UOK109 and 786-O cells was determined using CCK-8 assays after transfection for 48h. **(D-G)** A colony formation and tumor sphere formation assay were used to determine the colony and tumor sphere formation ability of UOK109 and 786-O cells co-transfected with indicated vectors, siRNA or miRNAs inhibitor. **(H-I)** The cell proliferation of UOK109 and 786-O cells were detected after co-transfection with indicated vectors, siRNA or miRNAs inhibitor by EdU. The data are presented as the mean ± SD, **P*< 0.05, ***P*< 0.01, ****P*< 0.001


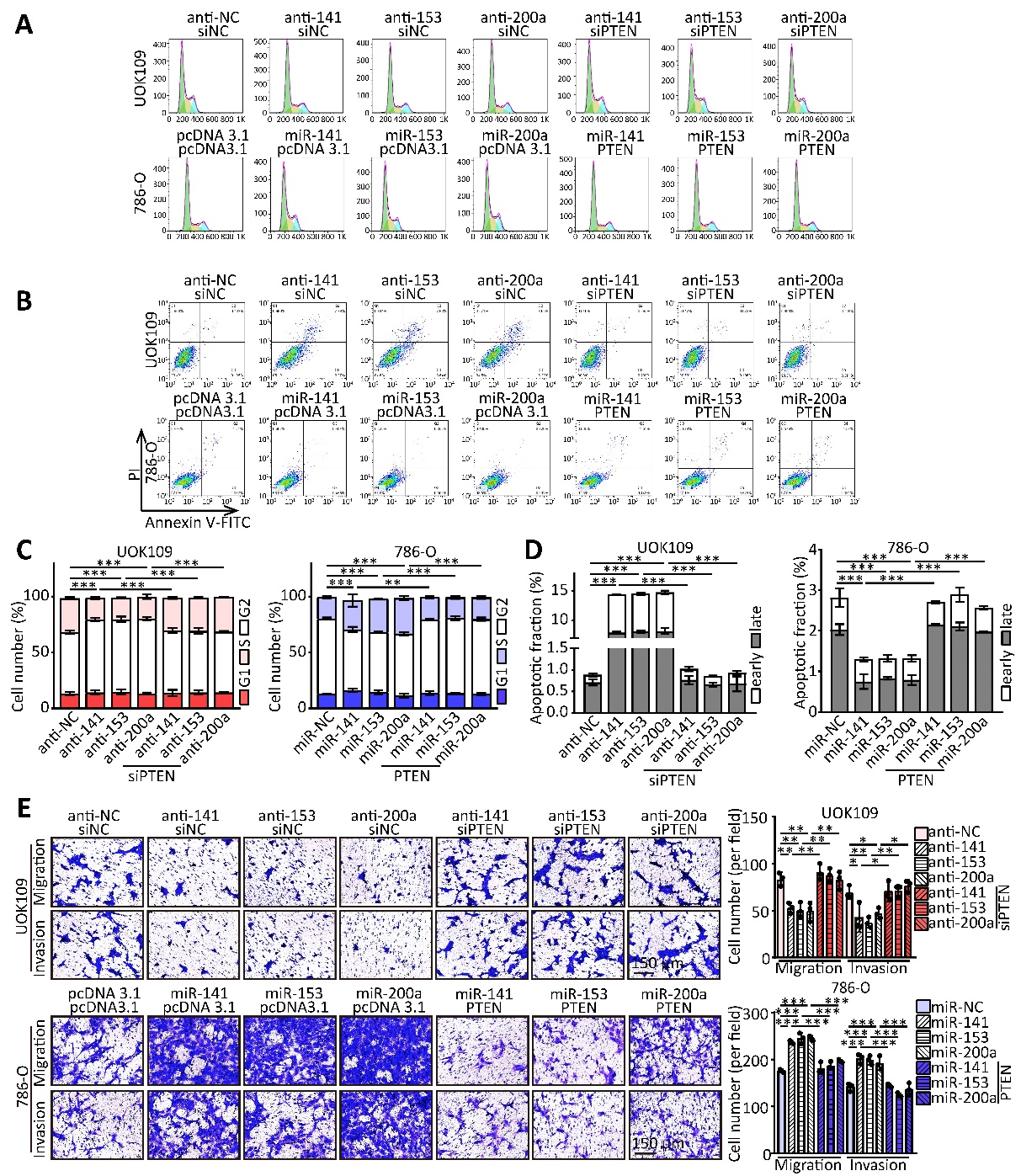


**Figure S17.** **Figure S14.** MiRNAs promote the cell cycle, apoptosis, migration and invasion of *NONO-TFE3* tRCC by targeting PTEN. **(A, C)** Cell cycle analysis was performed using flow cytometry in cells transfected with indicated vectors, siRNA or miRNAs inhibitor. **(B, D)** Apoptosis rate was tested using flow cytometry after transfection of UOK109 and 786-O cells for 48 h. **(E)** The invasion and migration abilities of UOK109 and 786-O cells co-transfected with indicated vectors, siRNA or miRNAs inhibitor were monitored by Transwell migration and invasion assays. The data are presented as the mean ± SD, **P*< 0.05, ***P*< 0.01, ****P*< 0.001


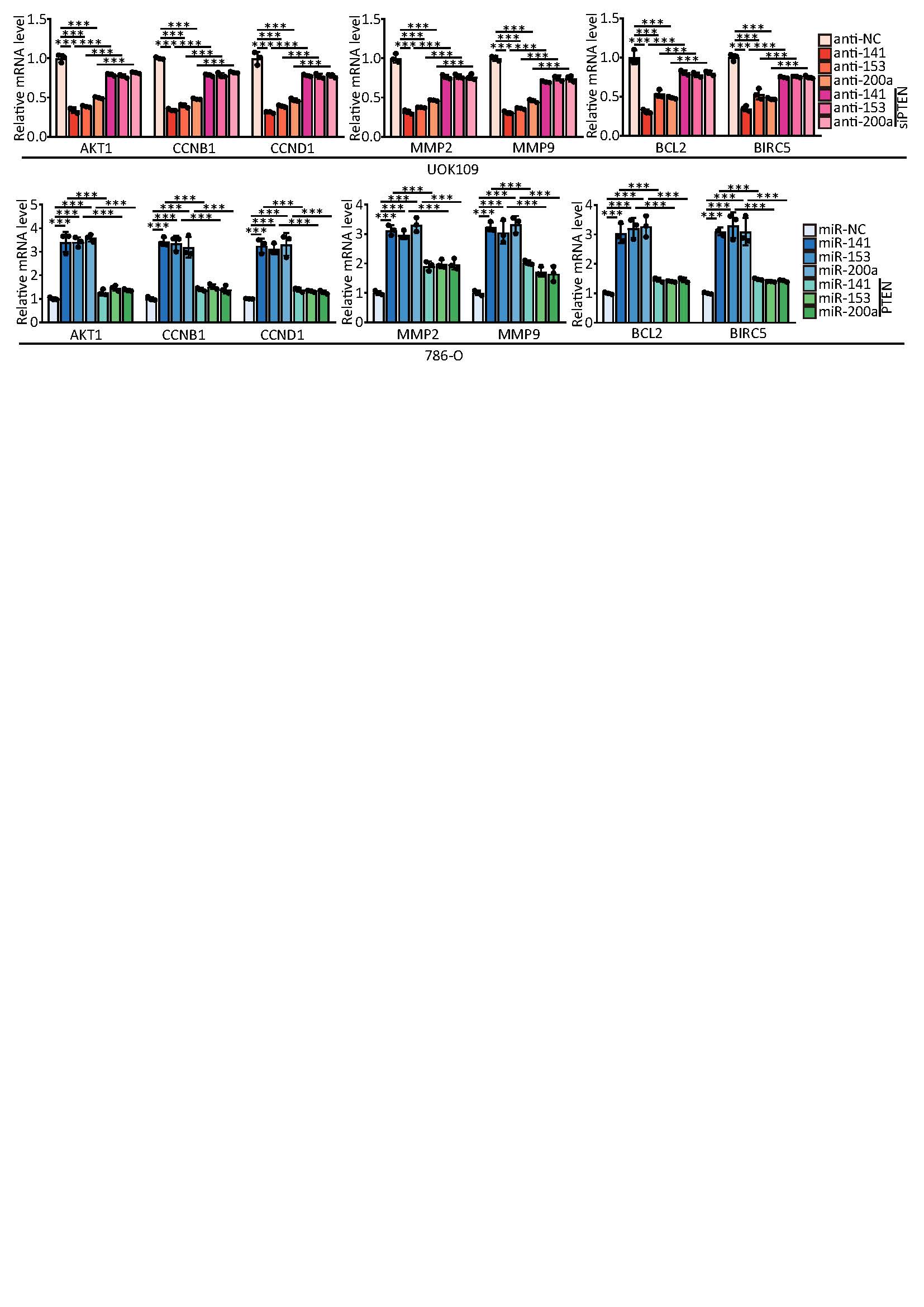


**Figure S18.** MiRNAs mediate the expression of genes related with cell proliferation, cell cycle, apoptosis, migration and invasion through PTEN in UOK109 and 786-O. The data are presented as the mean ± SD, ****P*< 0.001


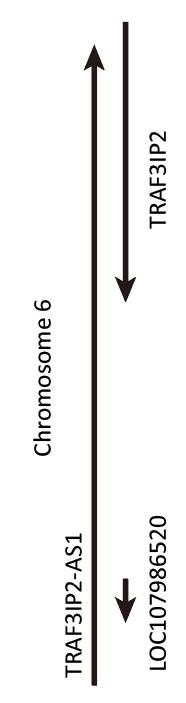

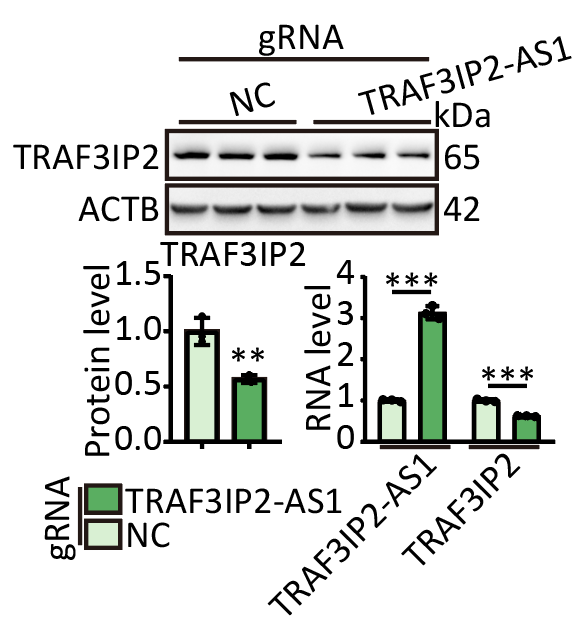


**Figure S19.** TRAF3IP2-AS1 suppressed the expression of TRAF3IP2 in UOK109. The data are presented as the mean ± SD, ***P*< 0.01, ****P*< 0.001


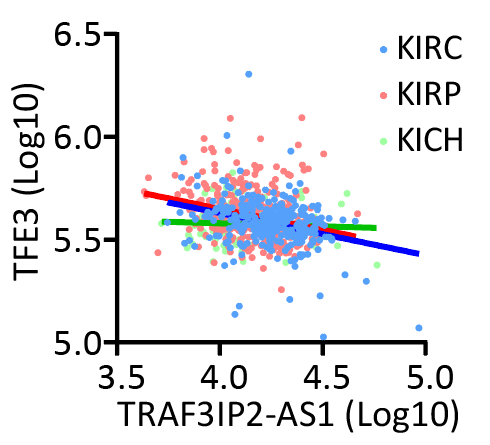


**Figure S20.** The correlation between TRAF3IP2-AS1 and wild type TFE3.


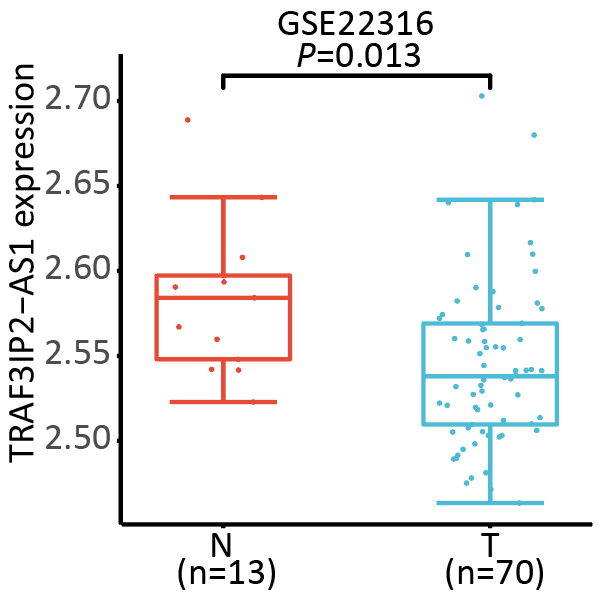


**Figure S21.** Analysis of TRAF3IP2-AS1 in ccRCC tissues compared with normal tissues was performed using GEO data (GSE22316).
